# Impaired insulin secretion via the Wnt5a/β-catenin pathway contributes to diabetes development in pancreatic cancer

**DOI:** 10.1038/s12276-025-01625-8

**Published:** 2026-01-28

**Authors:** Minyoung Lee, Ho Seon Park, Hyung Sun Kim, ARim Choi, Ji Hae Nahm, Beom Jin Lim, Jong Suk Park, Chul Woo Ahn, Younhee Ko, Dong Ki Lee, Dong Sup Yoon, Joon Seong Park, Shinae Kang

**Affiliations:** 1 Department of Internal Medicine, Yonsei University College of Medicine, Seoul, South Korea; 2 Institute of Endocrine Research, Yonsei University College of Medicine, Seoul, South Korea; 3 Department of Internal Medicine, Gangnam Severance Hospital, Yonsei University College of Medicine, Seoul, South Korea; 4 Department of Surgery, Pancreatobiliary Cancer Clinic, Gangnam Severance Hospital, Yonsei University College of Medicine, Seoul, South Korea; 5 Department of Pathology, Gangnam Severance Hospital, Seoul, South Korea; 6 Severance Institute for Vascular and Metabolic Research, Yonsei University College of Medicine, Seoul, South Korea; 7 Division of Biomedical Engineering, Hankuk University of Foreign Studies, Gyeonggi-do, South Korea; 8 Department of Surgery, Severance Hospital, Yonsei University College of Medicine, Seoul, South Korea; 9 Department of Surgery and Cancer Research Institute, Seoul National University Hospital, Seoul, South Korea

**Keywords:** Translational research, Pancreatic cancer

## Abstract

Diabetes is highly prevalent in individuals with pancreatic ductal adenocarcinoma (PDAC) and even precedes diagnosis of PDAC; however, the mechanisms of pancreatic cancer-associated blood glucose deterioration remain largely unknown. Here, we constructed a prospective cohort of patients undergoing pancreatectomy to investigate the underlying mechanism of PDAC-associated hyperglycemia. A total of 160 patients who underwent pancreatectomy (72 patients with PDAC and 88 patients without PDAC) were enrolled at a tertiary care hospital. Glucometabolic parameters under oral glucose tolerance test were assessed in both pre- and postoperative periods, and patient-derived blood and pancreatic tissue samples were collected. Compared with patients without PDAC, patients with PDAC showed severe hyperglycemia with impaired insulin secretion before surgery. However, despite identical type of pancreatectomy in both groups, hyperglycemia improved more significantly and insulin secretory function declined less after pancreatectomy in patients with PDAC. Plasma Wnt5a and pancreatic islet β-catenin levels were higher in patients with PDAC and correlated with the degree of hyperglycemia and insulin deficiency. Plasma Wnt5a levels also correlated with tumor size and pancreatic islet β-catenin expression in patients with PDAC. In rodent islets, Wnt5a treatment suppressed insulin release, which was recovered by inhibition of β-catenin. Collectively, impaired pancreatic insulin secretion by aberrant Wnt5a/β-catenin activation may underlie the hyperglycemia associated with PDAC. Our finding provides insights into the unique molecular mechanism of pancreatic cancer-associated hyperglycemia, paving the way for the identification of potential biomarker and therapeutic targets for this condition.

## Wnt5a/β-catenin activation impairs insulin secretion in patients with pancreatic cancer

Pancreatic ductal adenocarcinoma (PDAC) is a lethal cancer with low survival. Many patients with PDAC also develop diabetes, but the connection between the two is still unclear. Researchers aimed to explore this link by studying 160 patients, including those with PDAC and those without PDAC, who had surgery to remove part of their pancreas. The study involved measuring blood sugar and insulin levels before and after surgery. Patients with PDAC had higher blood sugar levels and lower insulin production than those without PDAC before surgery. After the surgery, patients with PDAC showed greater improvement in blood sugar control and a smaller decrease in insulin secretion than patients without PDAC. A protein called Wnt5a secreted by cancer was higher in patients with PDAC and might be linked to reduced insulin production. This protein could serve as a marker for early detection of PDAC-related diabetes.

This summary was initially drafted using artificial intelligence, then revised and fact-checked by the author.

## Introduction

Pancreatic ductal adenocarcinoma (PDAC) has a very poor survival rate, as most tumors are unresectable at diagnosis^1^. New-onset diabetes or worsening of pre-existing diabetes has been reported in a large proportion of patients with PDAC^1–3^, and possible pathophysiological associations between PDAC and incident diabetes have been described^4,5^. Therefore, studies to understand the missing link between PDAC and diabetes may enable early detection of PDAC^2^ and provide novel therapeutic targets for PDAC-associated diabetes.

Nevertheless, the distinct glucometabolic characteristics of PDAC-associated hyperglycemia have not been fully understood. A shortage of circulating insulin may partly explain hyperglycemia in PDAC^4^, but whether this reflects reduced β-cells or impaired insulin secretory function remains unclear. Importantly, there has not been adequate translational prospective human studies to investigate PDAC-associated metabolic features, partially because of the limited availability of biochemical samples from patients with PDAC with an appropriate surgical control group^6^.

To overcome these methodological limitations, we constructed a prospective cohort of 160 patients who underwent pancreatectomy. The database of the cohort contains information ranging from comprehensive glucometabolic parameters under oral glucose tolerance test (OGTT) to patient-derived blood samples and pancreatic tissues. Using this cohort, we investigated the changes in the phenotype of PDAC-associated diabetes before and after PDAC removal, as well as the underlying mechanisms with key molecules, thereby suggesting a potential biomarker for screening and tailored therapeutic targets for PDAC-induced diabetes.

## Materials and methods

### Study participants

We built a prospective cohort of 160 patients who underwent pancreatectomy, mainly pylorus-preserving pancreaticoduodenectomy (PPPD) (72 patients with PDAC and 88 patients without PDAC), at Gangnam Severance Hospital in Seoul, South Korea, from December 2014 to February 2019 (Supplementary Fig. 1a). The exclusion criteria were as follows: (1) diagnosis of neuroendocrine pancreatic tumor (*n* = 4), (2) pancreatic cancer other than adenocarcinoma (for example, mucoepidermoid carcinoma) (*n* = 3), and (3) steroid use before surgery (*n* = 1). Diabetes and prediabetes were defined according to the American Diabetes Association guidelines^7^. New-onset diabetes was defined as diabetes diagnosed less than 2 years before entry into the cohort, and recently aggravated diabetes was defined as the development of uncontrolled hyperglycemia less than 1 year before entry into the cohort in patients with known diabetes. The characteristics of the 160 patients are summarized in Supplementary Table 1. The human study protocol was approved by the independent institutional review board of Gangnam Severance Hospital, Seoul, South Korea (3-2014-0024).

### Mice

Healthy C57BL/6N male mice (8 weeks old) were housed in groups of three mice per cage, with free access to food and water under a strict 12-h light/dark cycle at a controlled temperature (23 ± 2 °C). All the animal experiments were approved by the Institutional Animal Care and Use Committee of Yonsei University Health System (YUHS-IACUC, 2019-0133).

### Cells

Insulinoma β-cell line, Min6 cells were maintained in Dulbecco’s modified Eagle medium (Gibco, Thermo Fisher Scientific/Gibco) supplemented with 10% fetal bovine serum and antibiotics (100 U/ml penicillin and 100 μg/ml streptomycin). These were originally received from Prof. Myung-Shik Lee, Yonsei University College of Medicine.

### Biochemical measurement of human participants

All patients underwent a 75 g OGTT just before and 2 weeks and 1 year after pancreatectomy. After an overnight fast, sampling was performed at 0, 30, 60, 90, and 120 min after oral intake of 75 g glucose to measure serum glucose and insulin levels. Serum biochemical parameters were evaluated after an overnight fast and 2 h after a normal meal. Serum glucose was measured by the glucose oxidase method using a 747 automatic analyzer (Hitachi). The levels of total cholesterol, triglyceride, and high-density lipoprotein cholesterol were quantified using an enzymatic colorimetric method (Hitachi 747, Daiichi). The low-density lipoprotein cholesterol content was calculated using the Friedewald formula^8^. Glycated hemoglobin A1c (HbA1c) levels were measured using high-performance liquid chromatography (Cobas Integra 800, Roche). Insulin and glucagon levels were measured using a radioimmunoassay method (Roche Diagnostics for insulin; MP Biomedicals for glucagon).

### Metabolic assessment of human participants

Hypertension was defined as systolic blood pressure (BP) of ≥140 mm Hg or diastolic BP of ≥90 mm Hg, current use of BP-lowering medication, or a self-reported history of hypertension. Dyslipidemia was defined as low-density lipoprotein cholesterol levels ≥160 mg/dl and/or triglyceride levels ≥200 mg/dl and/or high-density lipoprotein cholesterol levels <40 mg/dl^9^, or current use of lipid-lowering medication. Homeostasis model assessment of β-cell function (HOMA-β) and homeostatic model assessment of insulin resistance (HOMA-IR) were calculated as (360 × fasting insulin (μIU/ml))/(fasting glucose (mg/dl) − 63) and (fasting insulin (μIU/ml) × fasting glucose (mg/dl))/405, respectively^10^. The Matsuda index was defined as 10,000/√(fasting glucose × fasting insulin × mean glucose × mean insulin)^11^. The insulinogenic index was also calculated as (insulin (30 min) − insulin (0 min))/(glucose (30 min) − glucose (0 min))^12^. The disposition index obtained from the OGTT was used to evaluate the composite degree of insulin secretion in conjunction with insulin sensitivity and was calculated as the Matsuda index × insulinogenic index^13^.

### Pancreatic specimen collection

Specimens from human pancreas were obtained from Gangnam Severance Hospital (Seoul, South Korea) during the surgical procedure of pancreatectomy for histologic analysis. All pancreatic tissues were processed for formalin-fixed, paraffin-embedded and fresh-frozen pancreatic specimens.

### Mouse islet isolation and culture

Islets of 12–15-week-old C57BL/6 male mice were isolated by perfusion of the pancreas, followed by digestion using collagenase P solution in Hank’s balanced salt solution^14^. Islet cells were collected and cultured.

### GO enrichment analyses

In this study, we used a microarray dataset from the public database library of the Gene Expression Omnibus (GEO) associated with pancreatic cancer^15^ and diabetes^16^. Gene set enrichment analysis (GSEA) and Gene Ontology (GO) enrichment analysis were applied to the differentially expressed genes (DEGs) from these datasets. Gene sets were defined on the basis of GO and hallmark gene sets in the Molecular Signatures Database (MSigDB). GSEA was performed using the Java GSEA version 3.0 platform to generate enriched pathways, and enriched GO terms for DEGs were identified using a hypergeometric test.

## Results

### PDAC is significantly associated with worsened hyperglycemia and reduced insulin secretion

**Fig. 1:**
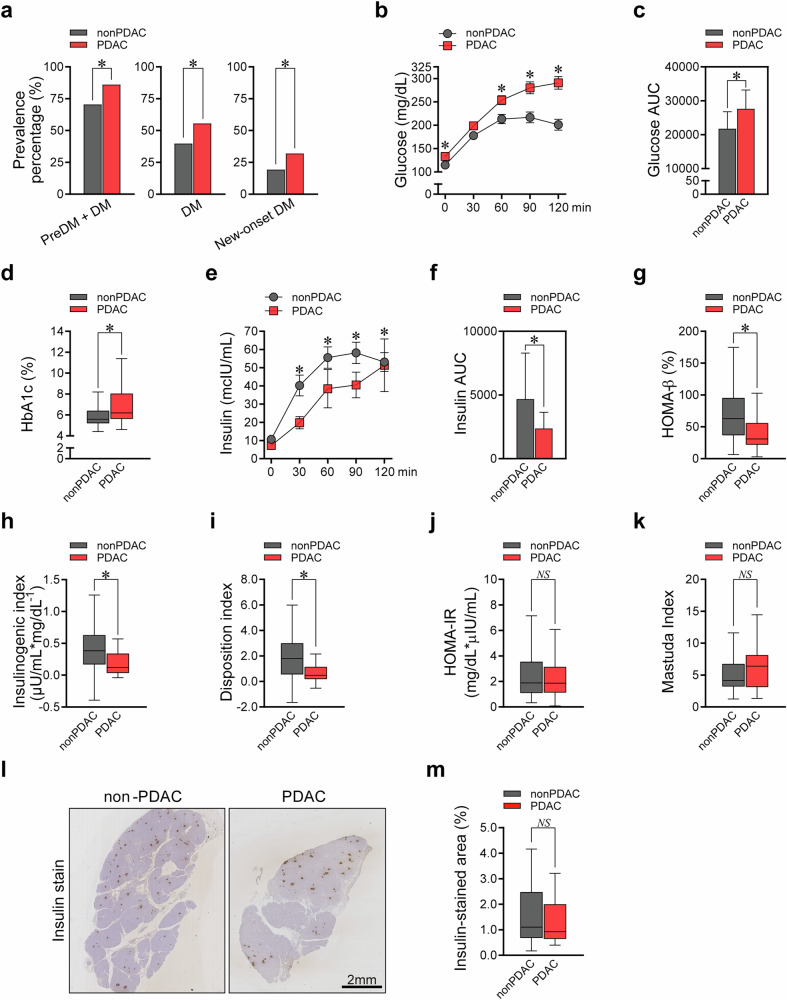
Glucometabolic characteristics of patients with PDAC versus patients without PDAC before surgery. **a**‒**k** The following parameters were compared between patients with PDAC and patients without PDAC before surgery: the prevalence of prediabetes and diabetes, diabetes, and new-onset diabetes (**a**); serum glucose levels during the 2-h OGTT (**b**); glucose AUC during the 2-h OGTT (**c**); HbA1c (**d**); serum insulin levels during the 2-h OGTT (**e**); insulin AUC during the 2-h OGTT (**f**); HOMA-β (**g**); insulinogenic index (**h**); disposition index (**i**); HOMA-IR (**j**); and Matsuda index (**k**). **l**, **m** Immunohistochemical staining on the pancreatic tissue of the resection margin obtained from PPPD surgery: representative insulin immunostaining image (magnification, 20×; scale bar, 2 mm) (**l**); quantification of the percentage of insulin-positive area in the total pancreatic area (**m**). All data are presented as medians with interquartile ranges, except for **a** (percentages) and **b** and **e** (means with standard error of the mean). *P <0.05 by chi-squared test (**a**), Mann–Whitney *U*-test (**b** except for differences in glucose at 60 and 90 min, **c**–**k,** and **m**), or Student’s *t*-test (differences in glucose at 60 and 90 min in **b**); NS, statistically nonsignificant.

Among the 160 patients enrolled prospectively in our pancreatectomy cohort, 88 were patients without PDAC and 72 were patients with PDAC (Supplementary Fig. 1a). The baseline characteristics and metabolic assessments before surgery are presented in Supplementary Table 1. The clinical diagnoses of patients in the non-PDAC group are summarized in Supplementary Fig. 1b. Compared with the patients without PDAC, patients with PDAC had a higher prevalence of a composite of prediabetes and diabetes (70.5% versus 86.1%), as well as diabetes (39.8% versus 55.6%), and new-onset (<2 years) diabetes (19.3% versus 31.9%) (Fig. 1a). Compared with the patients without PDAC, the patients with PDAC exhibited more severe hyperglycemia, higher fasting and postprandial glucose levels, greater glucose area under the curve (AUC) during OGTT, and elevated HbA1c (Fig. 1b–d). The PDAC group also exhibited significantly lower insulin secretory capacity, with reduced postprandial insulin, insulin AUC during OGTT, HOMA-β, insulinogenic index, and disposition index compared with the non-PDAC group (Fig. 1e–i). No difference was observed in insulin resistance-related parameters, such as HOMA-IR and Matsuda index (Fig. 1j, k). The histological analyses of surgical pancreatic specimens revealed no difference in insulin-stained β-cell area between the PDAC versus the non-PDAC groups (Fig. 1l, m). Altogether, hyperglycemia in patients with PDAC was mainly associated with the impaired insulin secretory function in patients with PDAC compared with patients without PDAC preoperatively.

There were no significant differences between the PDAC and non-PDAC groups in pre-existing metabolic comorbidities other than diabetes, or in nonglycemic metabolic indicators such as waist circumference, weight, body mass index (BMI), BP, and lipid profile (Supplementary Table 1). Therefore, the observed differences in glycemic and insulin secretion markers between the two groups were probably independent of underlying vulnerability to pre-existing metabolic disorders.

### Tumor removal recovers hyperglycemia and β-cell dysfunction in patients with PDAC

To address whether hyperglycemia and impaired insulin secretion were caused by pancreatic cancer cells in patients with PDAC, we evaluated the changes of glucometabolic parameters after ‘the intervention’ of tumor removal. To minimize the impact from the surgical procedure itself on blood glucose levels, we restricted analysis to those who underwent PPPD as their surgical method and enrolled patients without PDAC who underwent the same PPPD surgery as a control group. The comparison before and after surgery was analyzed specifically among 97 patients who underwent PPPD, out of a total of 160 patients who received any type of pancreatectomy.

**Fig. 2:**
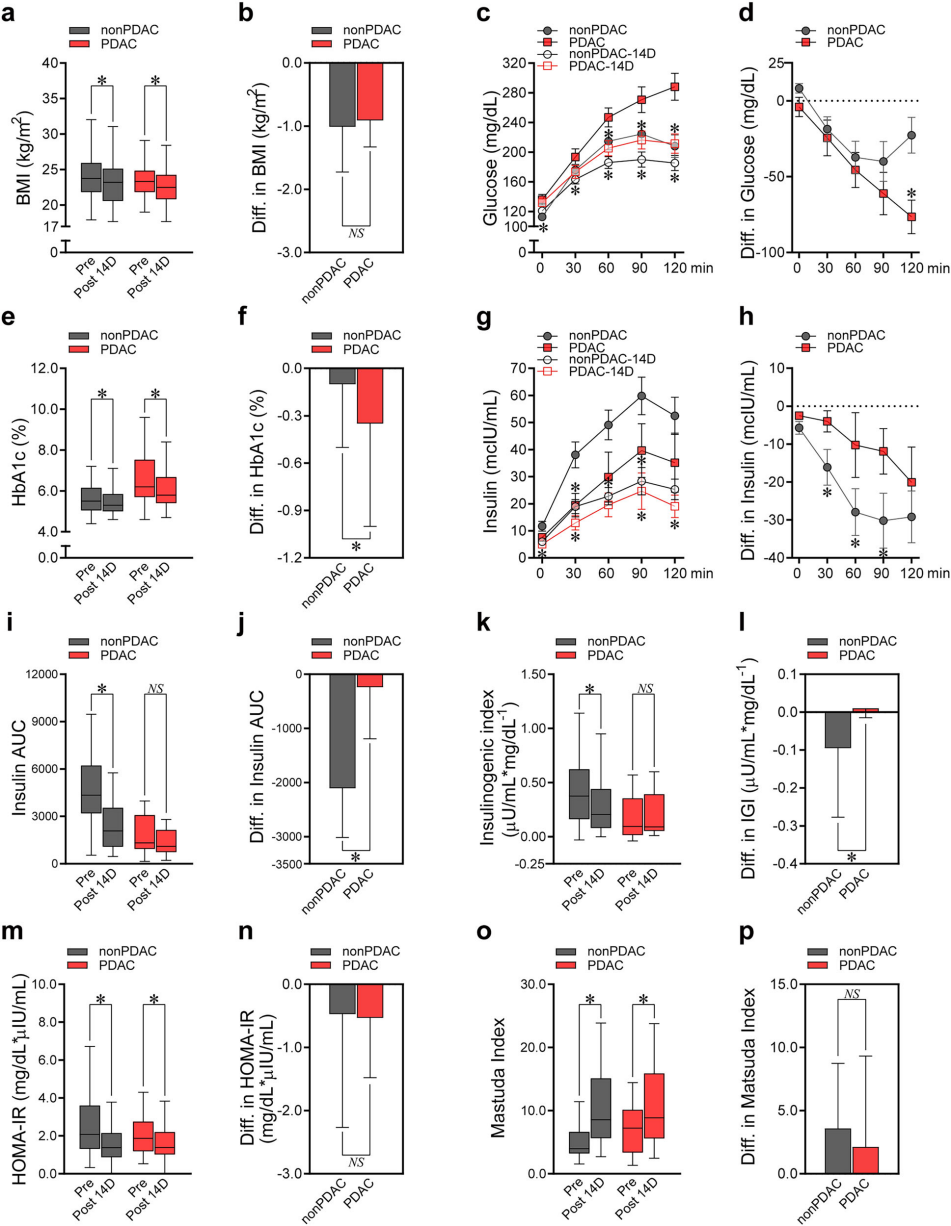
Different patterns of changes in glucometabolic parameters between patients with and without PDAC before and 14 days after surgery. Changes in the glucometabolic parameters between patients with and without PDAC before and 14 days after surgery. **a**, **b** BMI. **c**, **d**, Each point of serum glucose levels during the 2-h OGTT. **e**, **f** HbA1c. **g**, **h** Each point of serum insulin levels during the 2-h OGTT. **i**, **j** Insulin AUC during the 2-h OGTT. **k**, **l** Insulinogenic index. **m**, **n** HOMA-IR. **o**, **p** Matsuda index. In **a**, **c**, **e**, **g,** **i, k**, **m,** and **o**, glucometabolic parameters before PPPD were compared with those after PPPD for PDAC and non-PDAC groups. In **b**, **d**, **f**, **h**, **j**, **l**, **n,** and **p**, changes in glucometabolic parameters between before and 14 days after PPPD in the PDAC group were compared with those in the non-PDAC group. All data are presented as medians with interquartile ranges, except for **c**, **d**, **g**, and **h** (means with standard error of the mean). *P <0.05 by Wilcoxon signed-rank test (**a**, differences in glucose at 0 min in the PDAC group in **c**, **e,** **g** except for differences in insulin at 120 min in the non-PDAC group, **i**, differences in the PDAC group in **k**, **m**, and **o**), paired *t*-test (**c** except for differences in glucose at 0 min in the PDAC group, differences in insulin at 120 min in the non-PDAC group in **g**, and differences in the non-PDAC group in **k**), Mann–Whitney *U* test (**b**, differences in glucose at 0 min in **d**, **f**, **h**, **j**, **l**, **n**, and **p**), or Student’s *t*-test (**d** except for differences in glucose at 0 min); NS, statistically nonsignificant. IGI, insulinogenic index; Pre, preoperative status; Post 14D, postoperative status 14 days after surgery.

The changes in glucometabolic profiles at 14 days (Fig. 2 and Supplementary Table 2) and 1 year (Supplementary Fig. 2 and Supplementary Table 3) after PPPD are presented. At 14 days post-PPPD, the BMI decreased significantly in both the PDAC and non-PDAC groups (Fig. 2a), but the degree of BMI reduction did not significantly differ between the two groups (Fig. 2b). In both the PDAC and non-PDAC groups, the glucose levels during OGTT significantly decreased 14 days after surgery (Fig. 2c); however, the PDAC group showed a significantly greater reduction in postprandial glucose than the non-PDAC group (−23 mg/dl versus −61 mg/dl) (Fig. 2d and Supplementary Table 2). The HbA1c levels also decreased in both groups (Fig. 2e), but to a greater degree in the PDAC group (−0.1% versus −0.4%) (Fig. 2f).

At day 14 post-PPPD, the insulin levels during OGTT decreased significantly in both the patients wih PDAC and those without PDAC (Fig. 2g). However, the decline in insulin levels during OGTT was significantly less in the patients with PDAC (Fig. 2h). Although the PPPD procedure itself reduced insulin levels due to the loss of pancreatic volume common to both groups, the relatively smaller decrease observed in patients with PDAC compared with those without PDAC may be explained by partial restoration of insulin secretion following tumor removal in patients with PDAC, as insulin secretion had been suppressed before surgery. This is also supported by additional insulin secretory indices, such as the insulin AUC during OGTT and insulinogenic index, as these parameters significantly decreased only in the non-PDAC group but not in the PDAC group after PPPD (Fig. 2i–l and Supplementary Table 2). Improvements in HOMA-IR and the Matsuda index did not significantly differ between the PDAC and the non-PDAC groups (Fig. 2m–p). The disposition index significantly improved in the PDAC group, while there was no change in the non-PDAC group 14 days after PPPD (Supplementary Table 2). Both PDAC and non-PDAC groups showed a significant reduction in pancreatic volume after PPPD compared with preoperative measurements (Supplementary Table 2). However, there was no significant difference in the amount of volume reduction between the two groups (*P* = 0.318), suggesting the extent of pancreatic resection may not have caused differences in β-cell loss affecting insulin secretory capacity.

At 1 year post-PPPD, participants in both PDAC and non-PDAC groups showed a slight decrease in BMI without any difference between the groups (Supplementary Fig. 2a, b). In both PDAC and non-PDAC groups, the glucose levels decreased (Supplementary Fig. 2c), but the decrease in fasting glucose levels was more pronounced in the PDAC group than in the non-PDAC group (Supplementary Fig. 2d). In addition, the HbA1c level significantly decreased in the PDAC group 1 year after surgery but not in the non-PDAC group (Supplementary Fig. 2e, f). Interestingly, the insulin levels significantly decreased in the non-PDAC group, while they were relatively preserved in the PDAC group at 1 year post-PPPD (Supplementary Fig. 2g, h). Insulin secretory function, determined as HOMA-β, significantly improved in the PDAC group but not in the non-PDAC group (Supplementary Fig. 2i, j). Meanwhile, HOMA-IR did not change 1 year after PPPD in both the PDAC and non-PDAC groups (Supplementary Fig. 2k, l). Collectively, our data suggest a more persistent improvement in glycemic control in the PDAC group than in the non-PDAC group 1 year post-PPPD, which may be attributed to recovery of the insulin secretory function.

### Plasma Wnt5a level is increased in patients with PDAC and correlates with the degree of impairment in insulin secretion

**Fig. 3:**
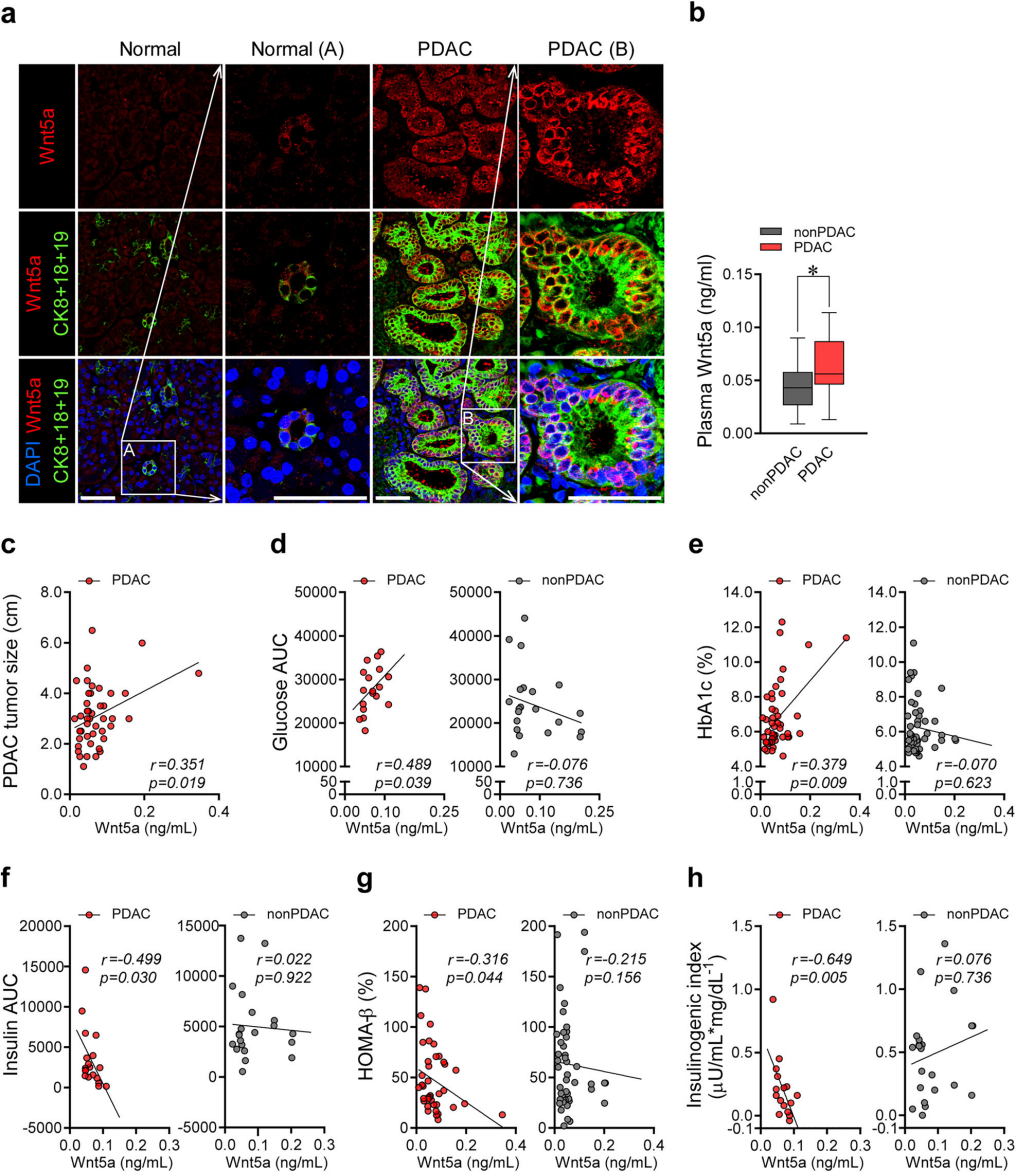
Plasma Wnt5a level and its association with glucometabolic parameters before surgery. **a** Pancreatic tissue was obtained from the cancer site of patients with PDAC and from the normal site of the patients without PDAC. Representative immunofluorescence staining images for Wnt5a (red) and Cytokeratin 8 + 18 + 19 (green), a marker for pancreatic ductal epithelial cells. Magnification, 400×, 1080×. Scale bar, 50 µm. **b**‒**h** Preoperative plasma Wnt5a levels measured by ELISA: difference in plasma Wnt5a level between PDAC and non-PDAC groups (**b**); correlation plots showing the associations between plasma Wnt5a and PDAC tumor size (**c**), glucose AUC during 2-h OGTT (**d**), HbA1c (**e**), insulin AUC during 2-h OGTT (**f**), HOMA-β (**g**), and insulinogenic index (**h**) in patients with and without PDAC. *P <0.05 by Mann–Whitney *U* test (**b**) or Spearman’s rank correlation coefficient (**c**–**h**); NS, statistically nonsignificant.

We analyzed the public microarray data from the tumor specimens of patients with PDAC and the islet tissue of patients with diabetes^15,16^ (Supplementary Fig. 3a, b) and found that the Wnt/β-catenin signaling may be a plausible pathway involved in PDAC-associated diabetes^17–21^. After preliminary testing of several molecules of the Wnt/β-catenin pathway using our cohort sample, we further investigated Wnt5a as a candidate molecule for PDAC-induced hyperglycemia^22–24^. Wnt5a was highly expressed in the tumor cells arising from the pancreatic duct in the patients with PDAC compared with the normal ductal cells from the patients without PDAC (Fig. 3a). Correspondingly, higher plasma levels of Wnt5a were observed in patients with PDAC (Fig. 3b). Furthermore, plasma Wnt5a levels significantly correlated with the primary tumor size, glucose AUC during OGTT, and HbA1c in patients with PDAC but not in patients without PDAC (Fig. 3c–e). The plasma Wnt5a levels also negatively correlated with insulin secretory parameters such as insulin AUC during OGTT, HOMA-β, and insulinogenic index in the PDAC group but not in the non-PDAC group (Fig. 3f–h). Interestingly, plasma Wnt5a levels also positively correlated with the serum levels of CA19-9, a widely used serum biomarker for pancreatic cancer^25^ (Supplementary Table 4).

We also evaluated the changes in plasma Wnt5a levels after tumor removal and its association with hyperglycemia and insulin secretion. At 14 days after PPPD, plasma Wnt5a levels were significantly decreased only in the PDAC group (Supplementary Fig. 4a). A greater decrease in plasma Wnt5a at 14 days after PPPD was associated with a larger reduction in HbA1c levels and greater improvement in the insulinogenic index in the PDAC group (Supplementary Fig. 4b, c). At 1 year after PPPD, a higher baseline plasma Wnt5a level was significantly associated with a greater reduction of fasting glucose and HbA1c levels and a greater improvement of HOMA-β in the PDAC group only (Supplementary Fig. 4d–f).

### Islet β-catenin expression correlates with the degree of hyperglycemia and impaired insulin secretory function in PDAC

**Fig. 4:**
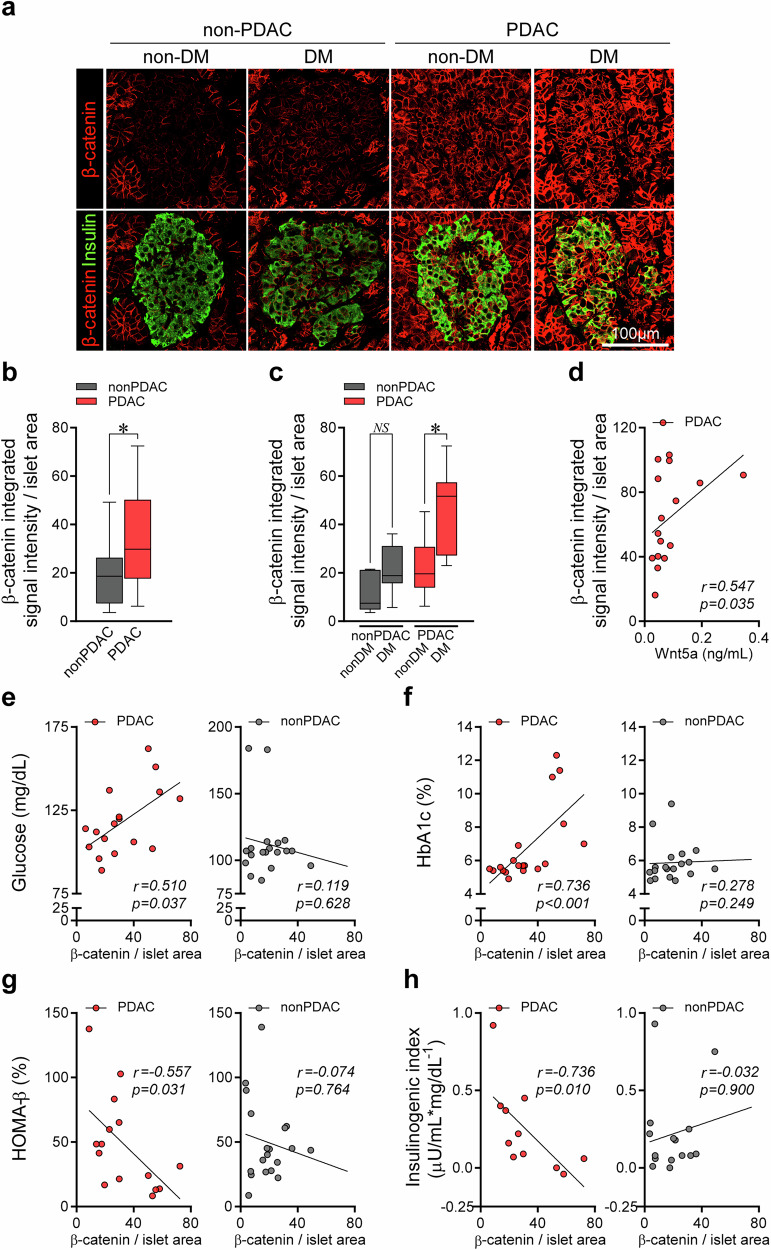
Islet β-catenin expression and its association with glucometabolic parameters before surgery. Pancreatic tissue from the same site of resection margin was obtained from patients with and without PDAC and then immunostained for β-catenin and insulin. **a** Representative immunofluorescence staining images for β-catenin (red) and insulin (green) in patients with and without PDAC, with and without diabetes. Magnification, 400×. Scale bar, 100 µm. **b**‒**h** The integrated signal intensity of β-catenin expression per islet area was calculated: difference of islet β-catenin expression between PDAC and non-PDAC groups (**b**); islet β-catenin expression according to diabetes status in PDAC and non-PDAC groups (**c**); correlation plots showing the associations between the islet β-catenin expression and plasma Wnt5a level (**d**), glucose (**e**), HbA1c (**f**), HOMA-β (**g**), and insulinogenic index (**h**) in patients with and without PDAC. *P <0.05 by Mann–Whitney *U* test (**b** and **c**) or Spearman’s rank correlation coefficient (**d**–**h**). NS, statistically nonsignificant; DM, diabetes mellitus.

To further elucidate the Wnt5a-related mechanism in PDAC-induced hyperglycemia, we evaluated the expression of β-catenin, a part of the canonical Wnt signaling pathway, in the pancreatic islets and analyzed the association between its expression level and various glucometabolic parameters. The identical resection margins of the pancreas from PPPD surgery, whether PDAC or non-PDAC, were used for histological analysis. Both exocrine and islet β-cell areas in the pancreas of patients with PDAC had a significantly higher expression of β-catenin than those of patients without PDAC (Fig. 4a, b). In high-magnification images assessing nuclear translocation associated with β-catenin activation^23^, β-catenin within 4′,6-diamidino-2-phenylindole (DAPI)-stained nuclei was more evident in PDAC tissues, particularly in the case accompanied by diabetes (Supplementary Fig. 5). Overall, β-catenin showed limited nuclear localization, which needs to be interpreted in the context of membrane-dominant original subcellular distribution and the fasting state at the time of pancreatic tissue collection during surgery, in which β-catenin tends to remain in the cytoplasm^21,26^. Patients with PDAC who also have diabetes showed an even higher expression of β-catenin than patients with PDAC without diabetes (Fig. 4c). In addition, the plasma Wnt5a level, which was increased in the patients with PDAC compared with patients without PDAC, exhibited a positive correlation with the degree of islet β-catenin expression in the PDAC group (Fig. 4d and Supplementary Table 5). The expression level of islet β-catenin was significantly associated with the levels of fasting glucose, glucose AUC during OGTT, and HbA1c in the PDAC group, whereas no such association was found in the non-PDAC group (Fig. 4e, f and Supplementary Table 5). The insulin secretory function assessed by insulin AUC during OGTT, HOMA-β, and insulinogenic index was negatively associated with islet β-catenin expression levels only in the PDAC group (Fig. 4g,h and Supplementary Table 5). Furthermore, the islet β-catenin expression was significantly associated with the primary tumor size in the PDAC group (Supplementary Table 5).

Alterations in β-catenin–independent, noncanonical Wnt signaling were evaluated in pancreatic islet β-cells by measuring c-Jun expression^27–30^ (Supplementary Fig. 6), and no significant difference in the c-Jun expression in islets was observed between the PDAC and non-PDAC groups (Supplementary Fig. 6b). In analyses within each group (PDAC and non-PDAC), diabetes status did not influence c-Jun expression (Supplementary Fig. 6c). Despite reports of noncanonical Wnt signaling activation in pancreatic cancer^27^, noncanonical Wnt signaling in β-cells does not appear to explain the diabetes occurring in pancreatic cancer.

### Wnt5a-driven β-catenin activation significantly suppresses insulin secretion in association with decreased E-cadherin expression

**Fig. 5:**
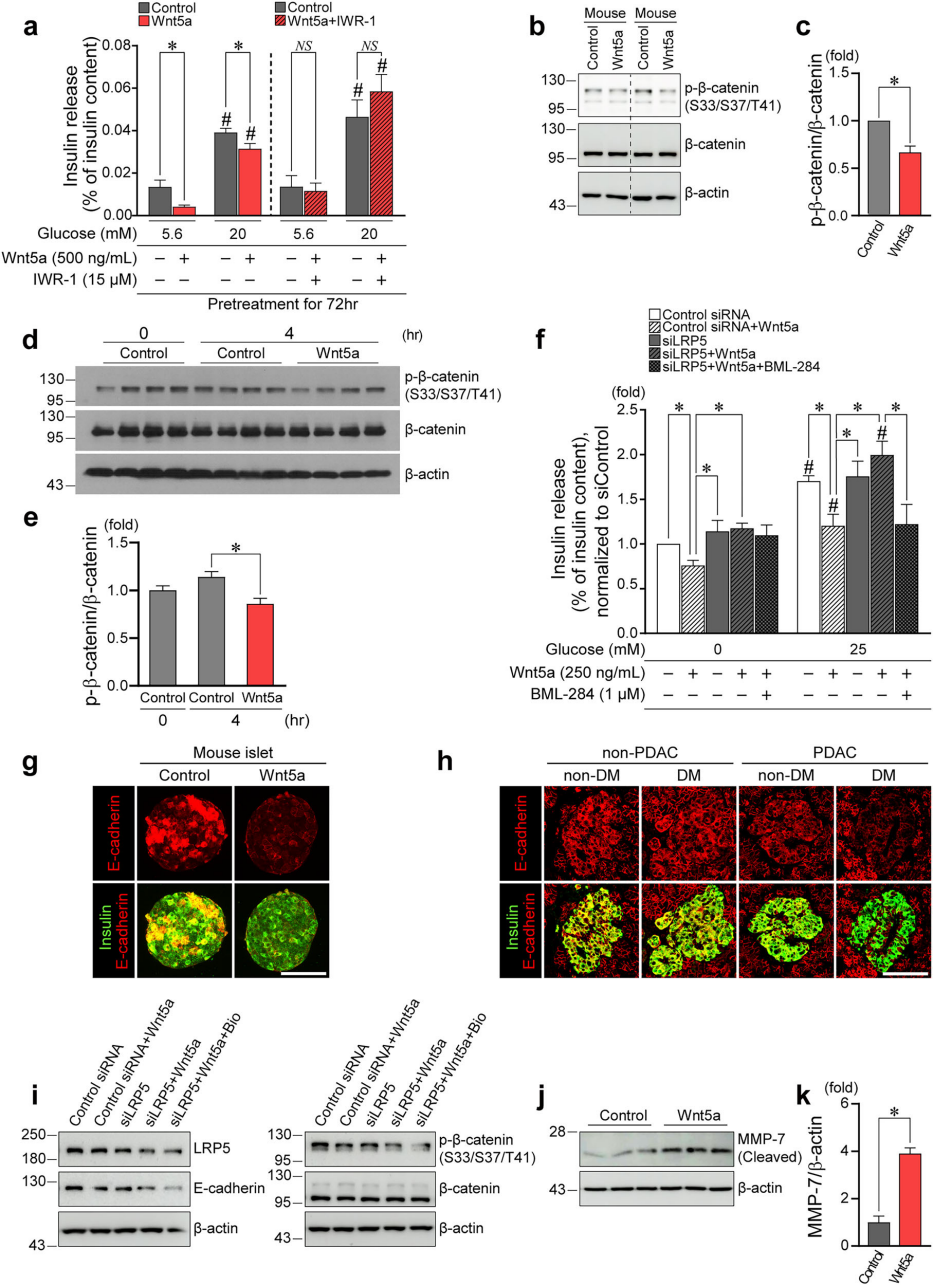
Wnt5a/β-catenin pathway-driven impaired insulin secretion and its association with decreased E-cadherin expression. **a** Degree of insulin release from isolated rodent islet cells in low (5.6 mM) and high (20 mM) glucose condition with/without treatment by Wnt5a (500 ng/ml for 72 h) and β-catenin antagonist, IWR-1 (15 µM for 72 h) (each group, *n* = 5). **b**‒**e** Western blotting for phosphorylated and total β-catenin after Wnt5a treatment (500 ng/ml for 4 h) in isolated rodent islets and MIN6 insulinoma cells (β-actin was used as a loading control): representative western blotting image of rodent islets (**b**); quantifications of phosphorylated β-catenin per total β-catenin in rodent islets (each group, *n* = 8) (**c**); representative western blotting image of MIN6 cells (**d**); quantifications of phosphorylated β-catenin per total β-catenin in MIN6 cells (**e**). **f** Insulin release from LRP5-knockdown MIN6 cells that were left untreated, treated with Wnt5a (250 ng/ml) alone, or cotreated with Wnt5a (250 ng/ml) and BML-284 (0.1 µM), under glucose-free (0 mM) and high-glucose (25 mM) conditions (each group, *n* = 3). Wnt5a and the Wnt signaling activator, BML-284, were treated 96 h before and 24 h after siRNA transfection. Insulin release was first calculated as the percentage of total insulin content and then normalized to siControl (= 1.0). **g**, **h** Isolated rodent islets and human pancreatic tissue were immunostained for E-cadherin (red) and insulin (green) (magnification, 400×; scale bar, 100 μm): representative immunofluorescence staining images in isolated rodent islets with/without Wnt5a treatment (500 ng/ml for 72 h) (**g**); representative immunofluorescence staining images in human pancreatic tissues from patients with and without PDAC, with and without DM (**h**). **i** Western blotting images of E-cadherin, phosphorylated β-catenin, and total β-catenin in LRP5-knockdown MIN6 cells. At 44 h post-siRNA transfection, cells were treated with Wnt5a (500 ng/ml) with and without BIO (1 µM) for 4 h. **j** Representative Western blotting image of MMP-7 with and without Wnt5a treatment (500 ng/ml for 4 h) in MIN6 cells. **k** Quantifications of MMP-7 per β-actin (each group, *n* = 4). *P <0.05 by Student’s *t*-test (comparisons between control and treatment groups in **a** and **e**, except the low-glucose control versus Wnt5a+IWR-1 comparison in **a,** and between-group comparisons in **f** and **k**) or Mann–Whitney *U* test (low-glucose control versus Wnt5a+IWR-1 comparison in **a** and **c**). ^#^*P* <0.05 by paired *t*-test (comparisons between low- and high-glucose conditions in **a** and **f**, excluding the control condition used as the comparator for Wnt5a+IWR-1 in **a**) or Wilcoxon signed-rank test (comparison between low- and high-glucose conditions in the control condition used as the comparator for Wnt5a+IWR-1 in **a**). NS, statistically nonsignificant; DM, diabetes mellitus; LRP5, low-density lipoprotein receptor-related protein 5.

To confirm Wnt5a-mediated suppression of insulin secretion ex vivo, we evaluated glucose-stimulated insulin secretion according to Wnt5a treatment in isolated rodent islets. Wnt5a treatment significantly reduced insulin release both at 5.6 mM and after stimulation with 20 mM glucose (Fig. 5a). Cotreatment with a β-catenin antagonist IWR-1 recovered the Wnt5a-induced insulin secretion impairment (Fig. 5a), implying that the β-catenin pathway mediates decreased insulin secretion by Wnt5a treatment. Wnt5a treatment also significantly decreased the ratio of phosphorylated/total β-catenin protein in rodent islets (Fig. 5b, c) and MIN6 insulinoma cells (Fig. 5d, e), suggesting an activation of the canonical Wnt/β-catenin pathway^23,31^. In MIN6 cells stimulated with 25 mM glucose (Fig. 5f), Wnt5a treatment significantly reduced insulin secretion in the siControl condition, whereas knockdown of low-density lipoprotein receptor-related protein 5 (LRP5), the canonical Wnt receptor for Wnt5a^32^, restored insulin secretion from the Wnt5a-induced decrease. With the addition of Wnt signaling activator, BML-284^33,34^, the rescue of insulin secretion mediated by LRP5 knockdown was reversed.

Considering that E-cadherin is involved in the vesicular secretion of insulin from pancreatic β-cells^35^ and is associated with β-catenin signaling^36,37^, we investigated the changes in cellular expression levels of E-cadherin by Wnt5a treatment. Immunofluorescence staining of rodent islets revealed that E-cadherin was co-expressed along with insulin, and Wnt5a treatment decreased E-cadherin and insulin expression (Fig. 5g). Immunofluorescence staining for E-cadherin and insulin in pancreatic tissues from patients with and without PDAC also demonstrated that the pancreatic expression of E-cadherin was generally decreased in patients with PDAC compared with that in patients without PDAC. Patients with PDAC who also have diabetes showed a more pronounced decrease in E-cadherin expression compared with patients with PDAC without diabetes (Fig. 5h). The link between Wnt5a-induced β-catenin activation and E-cadherin downregulation was further substantiated by LRP5 knockdown in the β-cell line MIN6, which attenuated Wnt5a signaling in the canonical β-catenin pathway (Fig. 5i). Under the LRP5 knockdown condition, Wnt5a treatment did not produce a marked reduction in E-cadherin. Notably, restoration of β-catenin activity with Wnt pathway activator BIO led to a marked decrease in E-cadherin even under LRP5 knockdown. Alterations in matrix metalloproteinase-7 (MMP-7) as a candidate mediator linking Wnt5a/β-catenin to E-cadherin downregulation were also examined^38–40^, and Wnt5a significantly upregulated MMP-7 in β-cells (Fig. 5j, k).

## Discussion

**Fig. 6:**
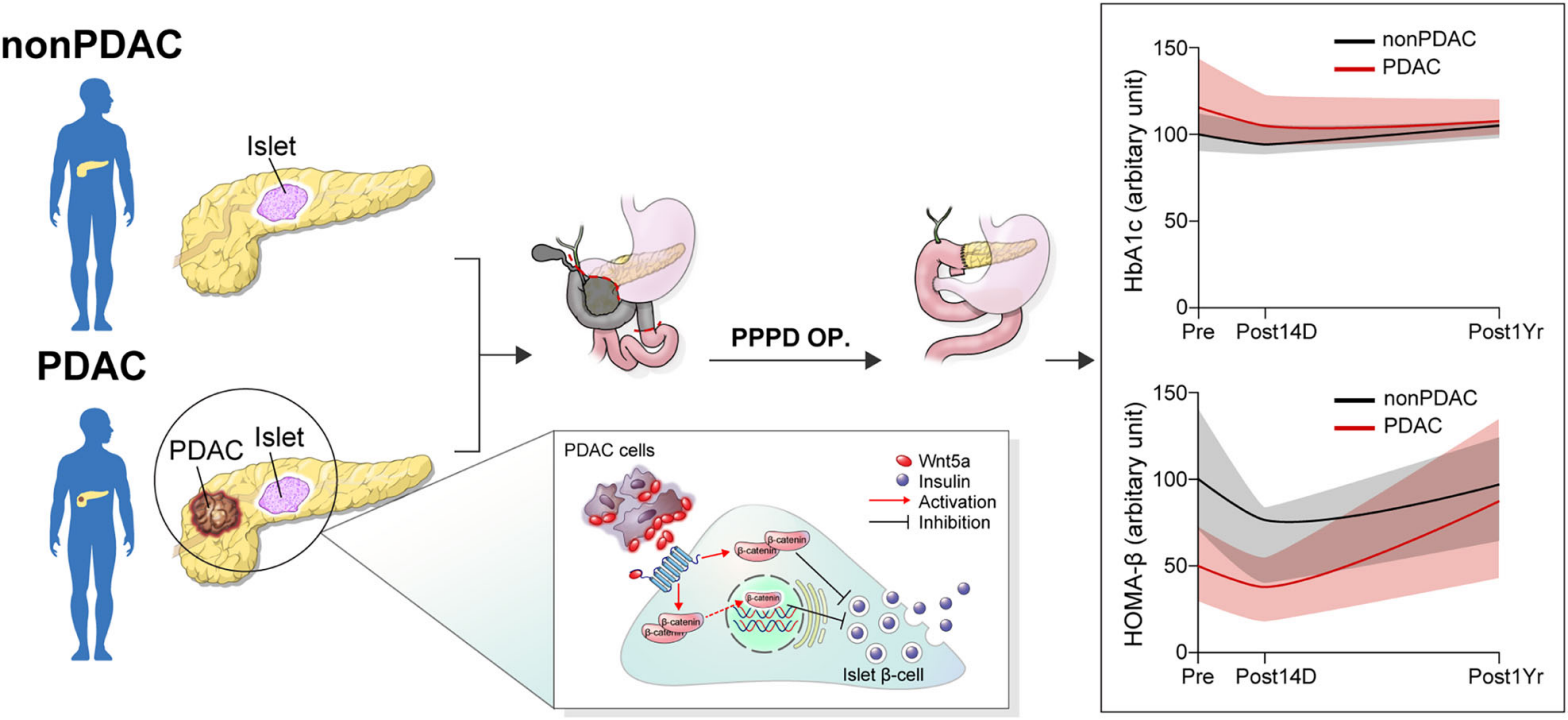
Graphical overview of PDAC-associated hyperglycemia and its pathophysiological mechanisms. Greater improvement in hyperglycemia after PPPD in patients with PDAC than in those without PDAC may be due to recovery from Wnt5a/β-catenin pathway-induced impairment of insulin secretion. OP operation.

New-onset diabetes before detection of pancreatic cancer is frequently observed, supporting a unique causal relationship between pancreatic cancer and diabetes^1–4^. Thus, studies on the pathophysiological mechanism to understand this relationship may enable early detection of PDAC that is preceded by hyperglycemia and identify potential therapeutic targets for PDAC-associated diabetes. In the current study, we observed that PDAC-associated diabetes was mainly attributable to decreased insulin secretion, a finding that was reversed by PDAC removal. The level of plasma Wnt5a and that of islet β-catenin expression were higher in patients with PDAC than in those without PDAC and correlated with the degree of hyperglycemia and impairments in insulin secretion. Ex vivo and in vitro, Wnt5a/β-catenin activation suppressed insulin secretion, possibly by decreasing the expression of E-cadherin. Altogether, impaired insulin secretion via the Wnt5a/β-catenin signaling pathway in the pancreatic islets may contribute to the development of diabetes in pancreatic cancer (Fig. 6).

While accumulating evidence supports the association between hyperglycemia and PDAC^6,24^, there has been debate over whether insulin resistance or insulin deficiency plays a more contributory role to this association^41^. Insulin resistance was consistently observed in patients with PDAC and resolved after cancer resection in previous reports^41^. However, in the present study, the unique glucometabolic profiles in patients with PDAC, distinct from patients without PDAC, were largely explained by an insulin secretory defect. Furthermore, although there was an improvement in the indices for insulin resistance in patients with PDAC after tumor removal, patients without PDAC also showed similar improvements in insulin resistance after PPPD. Therefore, the improvement in insulin resistance in patients with PDAC is probably attributable to the PPPD surgery itself rather than the tumor removal at least in this study cohort. In the present study, the deficient insulin secretion and its recovery after PDAC removal in the patients with PDAC were verified through multiple parameters, including the insulinogenic index measured using OGTT. This allowed delicate assessments of insulin deficiency in humans, which is one of the strengths of this study.

Despite several studies pointing to paraneoplastic evidence in PDAC-associated diabetes^42–44^, the majority are supported by animal or cell experiments, not by organized human studies. In search for possible paraneoplastic pathways of PDAC-associated diabetes, we analyzed commonly enriched gene pathways in both pancreatic cancer and diabetes using public microarray data from human tissue^15,16^. We primarily considered secretory proteins as candidates to identify novel blood biomarkers of PDAC, especially in those with new-onset or suddenly worsening diabetes. The Wnt pathway is an important mediator in a variety of metabolic diseases^45^ and is a well-known driver of PDAC development^21^. We assessed blood levels of several ligand proteins of Wnt signaling^45–47^, a commonly enriched pathway in both pancreatic cancer and diabetes in our analysis, and plasma Wnt5a levels were significantly higher in PDAC patients than in non-PDAC patients. The plasma Wnt5a level and pancreatic β-catenin expression were correlated with hyperglycemia and impaired insulin secretion in patients with PDAC. In this study, knockdown of the canonical Wnt receptor at cell membrane, LRP5, reversed the Wnt5a-induced reduction in insulin secretion, providing additional evidence that Wnt5a regulates insulin secretion via the β-catenin-mediated canonical Wnt pathway^32^. With additional BML-284 treatment, the effect of LRP5 knockdown was attenuated. BML-284 is a Wnt signaling activator that enhances nuclear β-catenin-dependent transcriptional activation^33,34^. Thus, Wnt5a’s impact on insulin secretion may proceed via the terminal step of the β-catenin signaling cascade, culminating in nuclear translocation and increased transcriptional activity. These findings may suggest that secreted Wnt5a from the PDAC is a potential diabetogenic factor that suppresses insulin secretion. Wnt5a/β-catenin signaling pathway needs further validation as a candidate of screening for PDAC-associated diabetes, which presents with a distinct insulin secretory dysfunction.

Cadherin molecules support the release of insulin vesicles^35^, and the loss of E-cadherin in β-cells impairs glucose-stimulated insulin secretion^48^. β-Catenin forms a complex with E-cadherin that promotes insulin vesicle pooling and trafficking, enabling insulin secretory granules to be released in response to glucose in β-cells^35^. However, the link between Wnt5a-induced β-catenin activation and E-cadherin downregulation in β-cells has not previously been addressed, especially in the pancreatic cancer microenvironment. Here, we suggested that decreased E-cadherin induced by Wnt5a and subsequent β-catenin-dependent canonical Wnt pathway activation could underlie insulin secretory defect in PDAC-associated diabetes. This phenomenon may be partly mediated by MMP-7 upregulation. In pancreatic cancer cells, β-catenin is associated with the overexpression of MMP-7^38^, which degrades E-cadherin, thereby weakening cell–cell junctions and facilitating tumor invasion and metastasis^39^. However, although MMP-7 expression has been detected in pancreatic β-cells^40^, there has been no evidence that its expression is modulated by Wnt signaling; this study demonstrates that Wnt signaling can increase MMP-7 expression in β-cells. Restoration of decreased E-cadherin mediated by aberrantly activated Wnt/β-catenin pathway could be a potential therapeutic strategy to ameliorate impaired insulin secretion-induced diabetes. Further mechanistic work dissecting MMP-7, E-cadherin, and β-cell insulin secretory function is also warranted.

We evaluated the metabolic outcomes after tumor removal at 14 days post-surgery and with an extended follow-up to 1 year among patients who underwent PPPD. The average duration of hospital stay after PPPD in Korea is around 14 days^49^, and the first analysis was conducted on the 14th day after surgery to observe early postoperative changes right before discharge. Differences in glycemic parameters including HbA1c were noted within a week after surgery^50^, suggesting that at least 1 week post-PPPD may be adequate for evaluating early metabolic alterations. The timing of the late postoperative follow-up was determined by considering the schedule of adjuvant chemotherapy and the overall survival. To minimize the influence of adjuvant chemotherapy on glycemic control, it was reasonable to ensure at least a 6-month interval after surgery^51,52^. Given that the median overall survival after PDAC resection is 1–2 years^51,52^ and the 1-year survival rate is approximately 70% ^51^, the 1-year mark was considered appropriate for follow-up in a substantial proportion of patients. Compared with the non-PDAC group, the PDAC group showed more severe hyperglycemia before surgery which was reversed even within 2 weeks after tumor removal and lasted for 1 year. The more pronounced glycemic improvement and preservation of insulin secretory function observed in the PDAC group compared with the non-PDAC group after PPPD may not be explained by differences in the pancreatectomy volume and the resulting β-cell loss or remnant intestine^53^. The PDAC and non-PDAC groups had comparable β-cell mass per unit area of the pancreas before surgery, and due to the standardized nature of the PPPD procedure performed in both groups^54^, there was no significant difference in the volume of pancreas resected between them. This supports our research hypothesis that specific molecules produced by PDAC may play important roles in impaired insulin secretion and the development of diabetes.

This study investigated the molecular link between PDAC and diabetes using patient-derived specimens and glucometabolic tests including OGTT, a gold-standard test for evaluating metabolic status. However, there are several limitations. First, postoperative metabolic features 1 year after PPPD were investigated with an insufficient number of patients. A larger number of patients with a longer follow-up duration would be helpful. Second, we addressed only Wnt5a as a candidate player in PDAC-associated diabetes. Other signaling molecules may also be involved and require further elucidation. Third, the detailed molecular mechanisms underlying Wnt5a pathway in PDAC-induced hyperglycemia should be evaluated using genetically engineered animal model in separate studies to specify therapeutic targets for PDAC-induced hyperglycemia. Fourth, the generalizability of these results to other populations may be limited, as this study was conducted in participants of Asian ethnicity.

In conclusion, using human samples from patients with pancreatic cancer and rodent islets/cells, we elucidated the underlying mechanisms of pancreatic cancer-associated diabetes. We evaluated the changes in glucometabolic profiles before and after tumor removal in patients with PDAC and compared them with those of an appropriate surgical control group: patients without PDAC who underwent the same PPPD surgery as the patients with PDAC. We demonstrated that hyperglycemia in pancreatic cancer can be explained by the aberrant Wnt5a/β-catenin activation-induced insulin secretory defect. Our findings highlight the potential of this pathway for developing novel blood biomarkers of PDAC-associated diabetes, as well as therapeutic targets to restore insulin secretion and reduce hyperglycemia in PDAC.

## Supplementary information

Supplementary Information

## Data Availability

Supplementary Fig. 3 was generated from the publicly available GEO database under accession codes GSE20966 and GSE16515. The remaining data can be found in the the Article or its Supplementary Information.
