# Supplementary material for: Impaired insulin secretion via the Wnt5a/β-catenin pathway contributes to diabetes development in pancreatic cancer
Source: Exp Mol Med. 2026 Jan 28;58(1):272–83. doi: 10.1038/s12276-025-01625-8 (PMC12868803; doi:10.1038/s12276-025-01625-8)
Supplement: Supplementary file 1 — Supplementary Information [file 12276_2025_1625_MOESM1_ESM.pdf]

KEY RESOURCES

Reagent or Resource	Source	Identifier
Antibodies		
Anti-β-Actin 1:30000	Sigma-Aldrich	Cat#A3854
Anti-β-Catenin 1:100-1:1000	Bethyl Laboratories	Cat#A302-010A
Anti-phospho-β-catenin(Ser33/37/Thr41) 1:1000	Cell Signaling Technology	Cat#9561
Anti-Cytokeratin 8+18+19	Abcam	Cat#ab41825
Anti-E-cadherin 1:50	Santa Cruz Biotechnology	Cat#sc31021
Anti-E-cadherin 1:1000	Cell Signaling Technology	Cat#3195
Anti-Insulin 1:500	Sigma-Aldrich	Cat#I2018
Anti-Insulin 1:500	Agilent/Dako	Cat#A0564
Anti-Insulin 1:100	Cell Signaling Technology	Cat#4590
Anti-Insulin 1:50	Thermo Fisher Scientific/Invitrogen™	Cat#PA1-26938
Anti-Wnt5a 1:50	Abcam	Cat#ab174963
Anti-c-Jun	Cell Signaling Technology	Cat#9165
Anti-LRP5	Cell Signaling Technology	Cat#5713
Anti-MMP7	Cell Signaling Technology	Cat#3801
FITC-conjugated donkey anti-mouse IgG	Jackson ImmunoResearch	Cat#715-095-150
Cy™3-conjugated donkey anti-rabbit IgG	Jackson ImmunoResearch	Cat#711-165-152
Cy™3-conjugated donkey anti-goat IgG	Jackson ImmunoResearch	Cat#705-165-147
Alexa Fluor® 488 AffiniPure® Donkey Anti-Guinea Pig IgG	Jackson ImmunoResearch	Cat#706-545-148
Donkey anti-Rabbit IgG (H+L) Highly Cross-Adsorbed Secondary Antibody, Alexa Fluor™ 594	Thermo Fisher Scientific/Invitrogen™	Cat#A-21207
Donkey anti-Rabbit IgG (H+L) Highly Cross-Adsorbed Secondary Antibody, Alexa Fluor™ 488	Thermo Fisher Scientific/Invitrogen™	Cat#A-21206
horseradish peroxidase-conjugated goat anti-mouse IgG	Vector laboratories	Cat#PI-200
Chemicals, Peptides, Recombinant Proteins and Tool		
BLOXALL solution	Vector laboratories	Cat#SP-6000
Citrate buffer	Agilent/Dako	Cat#S2369
Collagenase P	Roche	Cat#11213865001
DAPI(4',6-Diamidino-2-Phenylindole)	Thermo Fisher Scientific/Invitrogen™	Cat#D1306
Dulbecco's modified Eagle's medium (DMEM)	Thermo Fisher Scientific/Gibco™	Cat#41966029
Normal donkey serum	Jackson ImmunoResearch	Cat#017-000-121
endo-IWR-1	Tocris Bioscience	Cat#3532
Fetal bovine serum (FBS)	Thermo Fisher Scientific/Gibco™	Cat#16000044
Fluorescence Mounting Medium	Agilent	Cat#S3023
Glucose	Sigma-Aldrich	Cat#G7021
Hank's balanced salt solution	Thermo Fisher Scientific/Gibco™	Cat#14175095

HEPES buffer	Thermo Fisher Scientific/Gibco™	Cat#15630080
Lipofectamine™ RNAiMAX Transfection Reagent	Thermo Fisher Scientific/Invitrogen™	Cat#13778150
BIO	Sigma-Aldrich	Cat#B1686
BML-284	MedChemExpress	Cat#HY-19987
Perm washing buffer	Biolegend	Cat#421002
Chamber slide	Thermo Fisher Scientific /Thermo Scientific™	Cat#154534
Masson's Trichrome Stain		
Acid fuchsin	Sigma-Aldrich	Cat#857408
Aniline Bluet	SHOWA	Cat#0150-2331
Biebrich scalet	Sigma-Aldrich	Cat#198102
Bouin's solution	Sigma-Aldrich	Cat#HT10132
Ferric chloride solution	Sigma-Aldrich	Cat#12322
Glacial acetic acid	Sigma-Aldrich	Cat#8.18755
Hematoxylin	Sigma-Aldrich	Cat#H3136
Hydrochloric acid	Sigma-Aldrich	Cat#320331
Phosphomolybdic acid	JUNSEI	Cat#84235-0410
Phosphotungstic acid	JUNSEI	Cat#84220-0410
Penicillin/Streptomycin	Thermo Fisher Scientific/Gibco™	Cat#15140122
Phosphate-Buffered Saline (PBS)	Thermo Fisher Scientific/Gibco™	Cat#10010023
Protease inhibitor cocktail solution	GenDEPOT	Cat#P3100-001
Phosphatase inhibitor cocktail solution	GenDEPOT	Cat#P3200-001
Recombinant human/mouse Wnt5a	R&D Systems	Cat#645-WN-010
Roswell Park Memorial Institute(RPMI)-1640	Thermo Fisher Scientific/Gobco™	Cat#11879020
Triton X-100	Sigma-Aldrich	Cat#T8532
siRNA		
Negative Control siRNA	BIONEER	Cat#SN-1021
siLRP5	BIONEER	Cat#16873-2
Critical Commercial Assays		
3,30-Diaminobenzidine (DAB) substrate kit	Vector laboratories	Cat# SK-4100
Insulin ELISA kit	ALPCO.	Cat#80-INSMSH-E01
Wnt5a ELISA kit	Cloud-Clone Corp.	Cat#SEP549Hu
Deposited Data		
Publicly available microarray datasets from Mayo Clinic pancreatic tumor and normal samples	GEO database	GSE16515
Publicly available microarray datasets from human beta-cell enriched pancreatic tissue	GEO database	GSE20966
Experimental Models: Cell Lines		
Mouse: MIN6	N/A	RRID:CVCL_0431
Experimental Models: Organisms/Strains		
C57BL/6N male mouse	Orient	C57BL/6NCrljOri

Software and Algorithms		
Graphpad Prism 8	Graphpad	https://www.graphpad.com/
SPSS statistics 23.0	IBM Corporation	http://www.spss.com.hk/software/statistics/
ImageJ program version 1.8.9_112	National Institutes of Health	http://imagej.nih.gov/ij/
Java GSEA version 3.0	[1]	http://software.broadinstitute.org/gsea/index.jsp

SUPPLEMENTARY METHODS

Histologic analysis

The pancreas was fixed in 10% neutral buffered formalin for 48 h, and then embedded in paraffin. Tissue sections (4 μm) were prepared using a microtome (Reichert Scientific Instruments, Buffalo, NY, USA) and placed on glass slides. For staining, tissue sections were deparaffinized and rehydrated using xylene and ethanol. The degree of pancreatic fibrosis was assessed by Masson's trichrome staining. For immunohistochemical staining of insulin, antigen retrieval was performed in citrate buffer (pH 6.0). After endogenous peroxidase of the rehydrated tissue sections was inactivated with BLOXALL solution (SP-6000, Vector Laboratories, Burlingame, CA, USA) for 10 min at 22±2 °C, samples were blocked with 10% normal goat serum for 1 h. The tissues were then incubated overnight with mouse anti-insulin antibody (1:1000, I2018, Sigma-Aldrich, St. Louis, MO, USA) at 4 °C. After washing, the tissues were incubated with an appropriate secondary antibody (horseradish peroxidase-conjugated goat anti-mouse IgG, 1:200, PI-200, Vector Laboratories) for 2 h at room temperature. The sections were then incubated with 3,3-diaminobenzidine (DAB, SK-4100, Vector Laboratories) for 5 min and counterstained with hematoxylin. Stained sections were scanned using a slide scanner (Leica SCN400F, Leica Microsystems GmbH, Wetzlar, Germany), and the slide images were captured using an image viewer (SCN400image viewer version 2.2, Leica Microsystems CMS GmbH). Images were analyzed using the ImageJ program (version 1.8.9_112, http://imagej.nih.gov/ij/, NIH, Bethesda, MD, USA). After adjusting for the threshold within the section image, the percentage of the surface area above the threshold after staining with insulin was measured to determine the islet area. For immunofluorescence staining, the slides were incubated with anti-insulin antibodies (1:500, I2018, Sigma-Aldrich, St. Louis, MO, USA, 1:50, Thermo Fisher Scientific/Invitrogen™ ,

1:500, A0564, Agilent/Dako, 1:100, 4590, Cell Signaling Technology), rabbit anti-β-catenin antibody (A302-010A, 1:100, bethyl Laboratories, Montgomery, TX), rabbit anti-Wnt5a antibody (ab174963, 1:50, Abcam®, Cambridge, MA), mouse anti-Cytokeratin 8+18+19 (ab41825, 1:50, Abcam®), goat anti-E-cadherin (sc-31021 1:50, Santa Cruz biotechnology), or rabbit anti-c-Jun (1:400, 9165, Cell Signaling Technology) overnight at 4 °C. After washing three times for 5 min with PBS, the slides were incubated with FITC-conjugated donkey anti-mouse IgG (FITC AffiniPure Donkey Anti-Mouse IgG, Cat.# 715-095-150, 1:1000, Jackson ImmunoResearch, West Grove, PA), Alexa Fluor 488 dye-conjugated donkey anti-guinea pig IgG (Alexa Fluor® 488 AffiniPure® Donkey Anti-Guinea Pig IgG, Cat.# 706-545-148, 1:1000, Jackson ImmunoResearch), Cy™3-conjugated donkey anti-rabbit IgG (Cy™3 AffiniPure Donkey Anti-Rabbit IgG, Cat.# 711-165-152, 1:1000, Jackson ImmunoResearch), Cy™3-conjugated donkey anti-goat IgG antibody (Cy™3 AffiniPure Donkey Anti-goat IgG, Cat.# 705-165-147, 1:1000, Jackson ImmunoResearch), or Alexa Fluor 594 dye-conjugated donkey anti-rabbit IgG (Donkey anti-Rabbit IgG (H+L) Highly Cross-Adsorbed Secondary Antibody, Alexa Fluor™ 594, A-21207, Thermo Fisher Scientific/Invitrogen™) in blocking buffer for 30 min at room temperature, respectively. The sections were then washed and were stained with DAPI (4',6-Diamidino-2-Phenylindole) (DAPI, D1306, Thermo Fisher Scientific/Invitrogen™) and coverslipped with fluorescent mounting medium (Fluorescence Mounting Medium, S3023, Agilent). Fluorescent images were captured using a Zeiss Laser Scanning Confocal Microscope (LSM 780 or LSM980, Carl Zeiss, Oberkochen, Germany). All images were obtained using identical acquisition parameters as 12-bit, 1024×1024 arrays. Islet β-catenin and c-Jun-integrated signal density per islet area were measured and quantified using ImageJ software. For statistical analysis, every islet in each slide section was evaluated at ×200 magnification for β-catenin and at ×400 magnification for c-Jun: 38 patients for β-

catenin (non-PDAC without diabetes [n=8], non-PDAC with diabetes [n=11], PDAC without diabetes [n=8], PDAC with diabetes [n=11]) and 25 patients for c-Jun (non-PDAC without diabetes [n=7], non-PDAC with diabetes [n=5], PDAC without diabetes [n=6], PDAC with diabetes [n=7]).

Measurement of pancreatic volume using abdominopelvic computed tomography (APCT)

Pancreatic volume was measured using contrast-enhanced APCT images and analyzed with Aview software (version 1.1.44.30, Coreline, Korea). Axial CT images were first reformatted to generate multiplanar reformatted views, including all axial, sagittal, and coronal planes, as well as a 3D reconstruction of the pancreas. Window level and width were adjusted individually for each patient to optimize pancreatic visualization. Pancreatic contour was manually delineated on each slice using the brush tool while continuously referencing the sagittal and coronal views, as well as the real-time 3D rendering, to guide segmentation through ambiguous regions. Threshold-based segmentation was avoided because of the heterogeneous Hounsfield unit distribution within the pancreatic tissue. Contrast-enhanced vascular structures were excluded from the volume, while the pancreatic duct was included. After initial masking, the segmentation was reviewed and refined using a 3D sculpting function. Over-segmented areas outside the pancreatic border were removed, and under-segmented areas were supplemented to accurately reflect the anatomical boundaries. The final pancreatic volume was expressed in cubic centimeters (cc), equivalent to cm^3.

Enzyme-linked immunosorbent assay (ELISA)

Plasma levels of Wnt5a were quantified using ELISA kits (catalog #SEP549Hu, Cloud-Clone Corp., Houston, TX, USA) according to the manufacturer's instructions. For *ex vivo* and *in*

vitro analyses, the insulin levels in the culture supernatants were measured using ELISA kits (80-INSMSH-E01, ALPCO, Salem, NH, USA) according to the manufacturer's instructions.

***Ex vivo* assay of glucose homeostasis**

The islets were cultured overnight in RPMI-1640 (Thermo Fisher Scientific, Waltham, MA, USA) with 10% fetal bovine serum (Thermo Fisher Scientific), followed by maintenance with vehicle or recombinant human/mouse Wnt5a (500 ng/mL, 645-WN-010, R&D Systems, Minneapolis, MN, USA) or recombinant human/mouse Wnt5a (500 ng/mL) + β-catenin antagonist IWR-1 (15 μM, endo-IWR-1, 3532, Tocris Bioscience, Ellisville, MO, USA)-containing media for 72 h. Glucose-stimulated insulin secretion (GSIS) analysis of cultured islets was performed as previously described[2]. Evenly sized islets were selected for analysis (15 islets). These islets were incubated for 1 h with 2.8 mM glucose in Krebs Ringer Bicarbonate Buffer (KRBB) (118 mM $NaCl_2$, 4.7 mM KCl, 2.5 mM $CaCl_2$, 1.18 mM KH_2PO_4, 1.18 mM $MgSO_4$, 25 mM $NaHCO_3$, 10 mM HEPES, 0.1% BSA) and then stimulated with 5.6 or 20 mM of glucose each for 1 h. The supernatant from each incubated buffer was collected, and the amount of secreted insulin was quantified. The same set of islets was used to measure the total insulin content in the islets. Briefly, the islets were incubated for 24 h in 75% acidic ethanol containing 0.2 M HCl at 4 °C, and the supernatant was processed for analysis. The degree of GSIS was presented as percentage release, calculated by dividing the amount of secreted insulin in the KRBB by the total insulin content in the same batch of islets.

***Ex vivo* assay of E-cadherin**

Islets isolated from the same mouse were divided into a control group and a Wnt5a-treated group (500 ng/ml, 72 h). Wnt5a-treated islets were fixed in 4% paraformaldehyde for 1 h,

followed by immunofluorescence staining. The islets were permeabilized on ice for 15 min in microtubes using Perm Buffer (421002, BioLegend, San Diego, CA), blocked with 10% normal donkey serum for 1 h, and incubated overnight at 4 °C with goat anti-E-cadherin (1:50, Santa Cruz Biotechnology) and rabbit anti-insulin (1:50, Cell Signaling Technology) antibodies. Subsequently, islets were incubated for 2 h with Cy™3-conjugated donkey anti-goat IgG (1:200, Jackson ImmunoResearch) and Alexa Fluor 488-conjugated donkey anti-rabbit IgG (1:200, A-21206, Thermo Fisher Scientific/Invitrogen™), mounted on chamber slides (154534, Thermo Fisher Scientific, Rochester, NY), and imaged using a confocal microscope.

Small interfering ribonucleic acid (siRNA) transfection

siRNAs were purchased from Bioneer (Control siRNA, Cat.SN-1021, siLRP5, Cat.16973-2, Daejeon, Korea). Min6 cells were transfected with siRNA using Lipofectamine RNAiMAX (Thermo Fisher Scientific/ Invitrogen™, Carlsbad, CA, USA) according to the manufacturer's protocol. Briefly, siRNA (final concentration: 66 nM) and Lipofectamine RNAiMAX were diluted in DMEM medium (Gibco) and incubated for 20 min at room temperature to allow complex formation. The complexes were then added to cells in antibiotic-free medium, which was replaced with fresh complete medium 6 h after transfection.

To restore β-catenin activity from the LRP5 knockdown, BML-284 and 6-bromo-indirubin-3'-oxime (BIO) was used as Wnt signaling activators. BML-284 facilitates the nuclear transcriptional co-activation of β-catenin with T cell factor, independently of glycogen synthase kinase-3 beta (GSK-3β) axis[3,4]. BIO is a GSK-3β inhibitor that prevents the phosphorylation of β-catenin by GSK-3β and subsequent degradation, which allows β-catenin to accumulate and be transported into the nucleus to drive transcription of Wnt target genes[5].

In vitro assay of glucose homeostasis

MIN6 cells were pretreated with Wnt5a (250 ng/mL) alone or in combination with BML-284 (0.1 μM), and the medium was replaced every 2 days with either Wnt5a (250 ng/mL) alone or Wnt5a (250 ng/mL) combined with BML-284 (0.1 μM). On day 4 of pre-treatment, cells were transfected with control siRNA or siLRP5 for 6 h, followed by replacement of the medium with either Wnt5a (250 ng/mL) alone or Wnt5a (250 ng/mL) combined with BML-284 (0.1 μM). GSIS was then performed 24 h after siRNA transfection (i.e., at 24 h under no treatment, Wnt5a, or Wnt5a + BML-284 conditions). MIN6 cells were washed twice with PBS and pre-incubated in serum-free, glucose-free RPMI-1640 medium for 1 h. Following pre-incubation, cells were sequentially incubated for 30 min in glucose-free (0 mM) RPMI-1640 medium, and then for 30 min in high-glucose (25 mM) RPMI-1640 medium. Supernatants were collected after each incubation period, and cells were harvested for determination of total insulin content. GSIS was calculated as the ratio of insulin released into the medium to the total insulin content of the corresponding cell batch.

Western blot analysis

Islets isolated from each individual mouse were divided into two portions and cultured as control islets or Wnt5a-treated islets (500 ng/mL) in RPMI-1640 medium containing 20 mM glucose for 4 h. MIN6 cells were stimulated by Wnt5a with or without BIO for the indicated duration. Subsequently, islets and MIN6 cells were washed and lysed in lysis buffer containing protease inhibitor and phosphatase inhibitor cocktail. Western blot was performed as previously described[6] with anti-phospho-β-catenin (Anti-phospho-β-catenin (Ser33/37/Thr41), Cat.9561, 1:1000, Cell Signaling Technology, Danvers, MA, USA), anti-β-catenin (Cat.A302-010A, 1:1000, bethyl laboratories, Montgomery, TX, USA), anti-LRP5 (Cat.5713, 1:1000, Cell

Signaling Technology), anti-E-cadherin (Cat.3195, 1:1000, Cell Signaling Technology), anti-MMP-7 (Cat.3801, 1:1000, Cell Signaling Technology), and anti-β-actin (Cat.A3854, 1:30000, Sigma Aldrich, St Louis, MO, USA).

Statistical analysis

Data for continuous variables are presented as mean with standard deviation (SD) or standard error of the mean (SEM) or as median with interquartile range (IQR), and data for categorical variables are presented as number with percentage. The Shapiro-Wilk test was used to determine whether the data was normally distributed. When comparing continuous variables between the two groups, statistical significance was tested using the Student's *t*-test for normal distributions or Mann Whitney *U*-test for non-normal distributions. Comparisons between pre- and post-operative clinical data or pre- and post-treatment experimental data were conducted using the paired *t*-test for normal distributions or Wilcoxon signed-rank test for non-normal distributions. Categorical variables were compared using the chi-square test or Fisher's exact test. Spearman's correlation coefficient was used to estimate the degree of correlation between the β-catenin or Wnt5a expression levels and glucometabolic parameters. *P*-values of <0.05 were considered statistically significant. All statistical analyses were conducted using SPSS software version 29.0 (IBM Corp., Armonk, NY, USA).

REFERENCES

1. Subramanian, A. et al. Gene set enrichment analysis: a knowledge-based approach for interpreting genome-wide expression profiles. *Proc Natl Acad Sci U S A* **102**, 15545-15550 (2005).

2. Park, H. S. et al. beta-Cell-Derived Angiopoietin-1 Regulates Insulin Secretion and Glucose Homeostasis by Stabilizing the Islet Microenvironment. *Diabetes* **68**, 774-786 (2019).

3. Liu, J., Wu, X., Mitchell, B., Kintner, C., Ding, S. & Schultz, P. G. A small-molecule agonist of the wnt signaling pathway. *Angew Chem Int Edit* **44**, 1987-1990 (2005).

4. Ahadome, S. D., Zhang, C., Tannous, E., Shen, J. & Zheng, J. J. Small-molecule inhibition of Wnt signaling abrogates dexamethasone-induced phenotype of primary human trabecular meshwork cells. *Exp Cell Res* **357**, 116-123 (2017).

5. Law, S. M. & Zheng, J. J. Premise and peril of Wnt signaling activation through GSK-3β inhibition. *iScience* **25**, 104159 (2022).

6. Choi, J. W. et al. BNIP3 is essential for mitochondrial bioenergetics during adipocyte remodelling in mice. *Diabetologia* **59**, 571-581 (2016).

SUPPLEMENTARY FIGURES

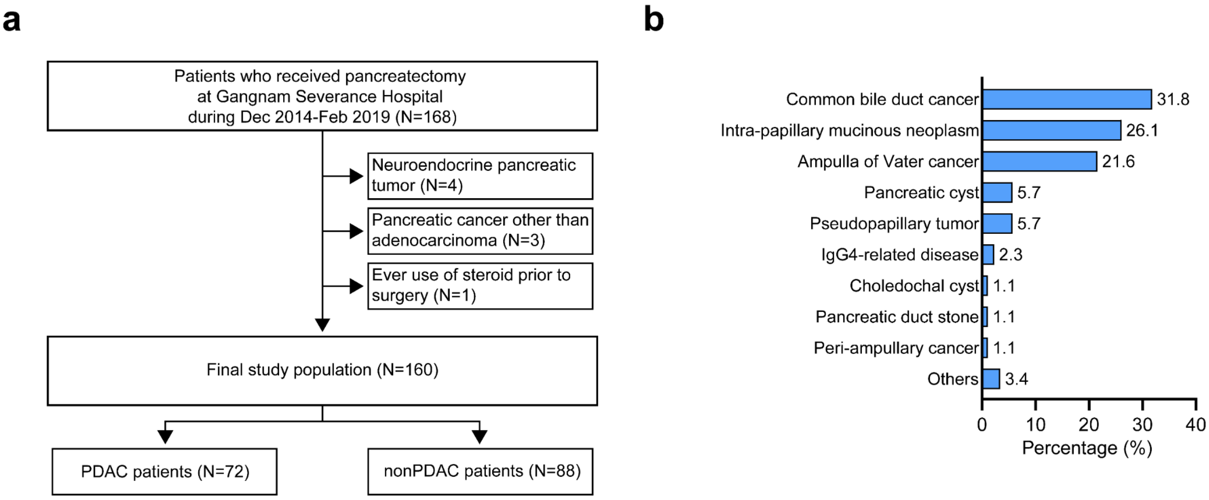

Supplementary Fig. 1 Study design and clinical diagnoses of study population

a Flowchart showing the selection process of study subjects. **b** Composition of diagnoses in non-PDAC subjects.

PDAC, pancreatic ductal adenocarcinoma.

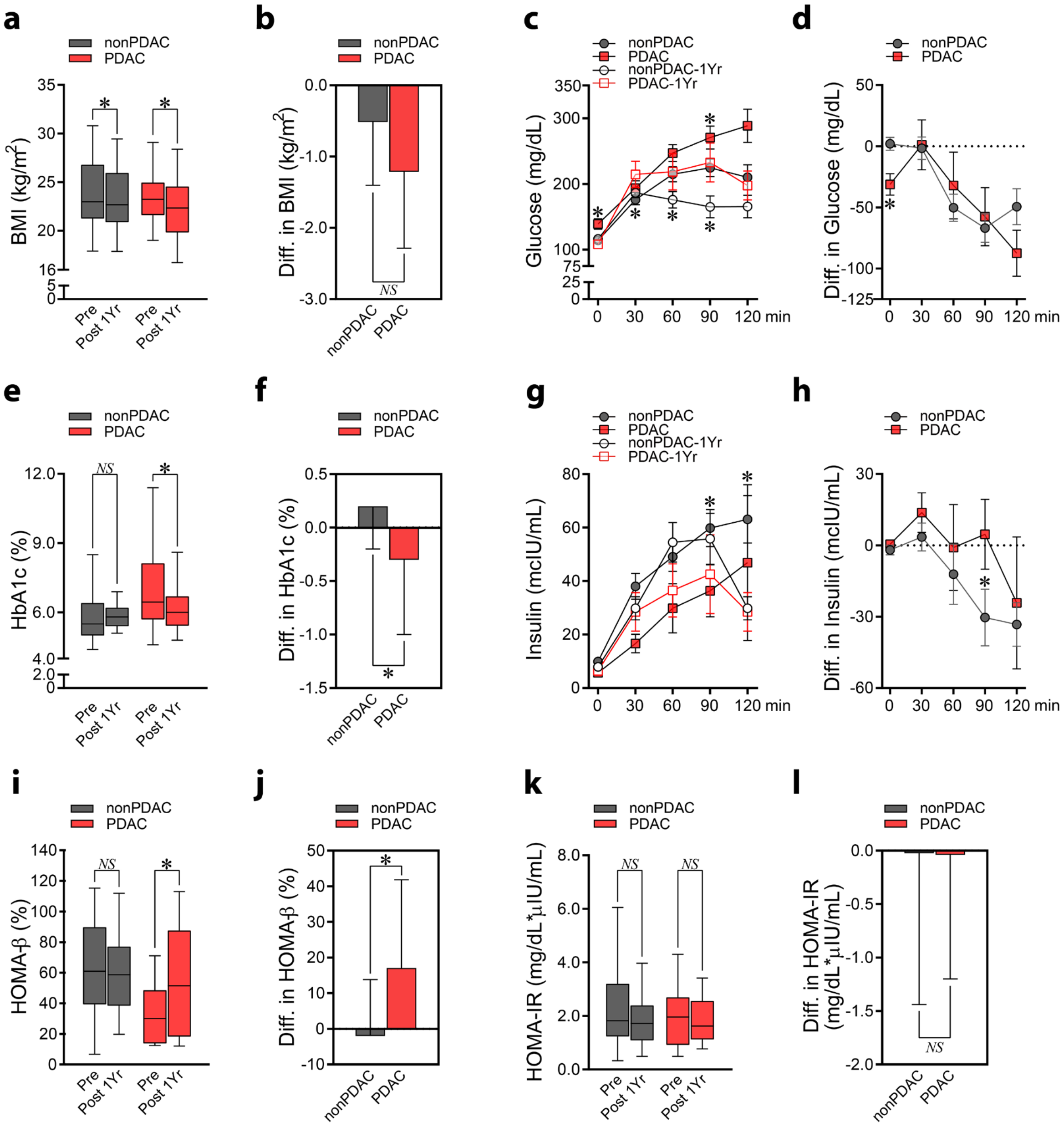

Supplementary Fig. 2 Different patterns of changes in glucometabolic parameters between patients with and without PDAC before and 1 year after surgery

Changes in the glucometabolic parameters between patients with and without PDAC before and 1 year after surgery. **a**, **b** BMI. **c**, **d** Each point of serum glucose levels during the 2-h OGTT. **e**, **f** HbA1c. **g**, **h** Each point of serum insulin levels during the 2-h OGTT. **i**, **j** HOMA-β. **k**, **l** HOMA-IR. In **a**, **c**, **e**, **g**, **i**, and **k**, glucometabolic parameters before PPPD were compared

with those after PPPD for PDAC and non-PDAC groups. In **b**, **d**, **f**, **h**, **j**, and **l**, changes in glucometabolic parameters between before and 1 year after PPPD in the PDAC group were compared with those in the non-PDAC group. All data are presented as medians with interquartile ranges, except for **c**, **d**, **g**, and **h** (means with standard error of the mean).

*P <0.05 by Wilcoxon signed-rank test (differences in the non-PDAC group in **a**, differences in glucose at 0 min in the PDAC and non-PDAC groups in **c**, **e**, **g** except for differences in insulin at 0 min in the PDAC group, insulin at 30 min in the PDAC and non-PDAC groups, and insulin at 60 min in the non-PDAC group, **i**, and **k**), paired *t*-test (differences in the PDAC group in **a**, **c** except for differences in glucose at 0 min in the PDAC and non-PDAC groups, differences in insulin at 0 min in the PDAC group, insulin at 30 min in PDAC and non-PDAC groups, and insulin at 60 min in the non-PDAC group in **g**), Mann Whitney *U*-test (**b**, **d** except for differences in glucose at 0 min, **f**, **h** except for differences in insulin at 30 min, **j**, and **l**), or Student's *t*-test (difference in glucose at 0 min in **d** and difference in insulin at 30 min in **h**); *NS*: statistically non-significant.

AUC, area under the curve; BMI, body mass index; HbA1c, glycated hemoglobin A1c; HOMA-β, homeostasis model assessment for β-cell function; HOMA-IR, homeostatic model assessment for insulin resistance; OGTT, oral glucose tolerance test; PDAC, pancreatic ductal adenocarcinoma; PPPD, pylorus-preserving pancreaticoduodenectomy; Pre, pre-operative status; Post 1Yr, post-operative status 1 year after surgery.

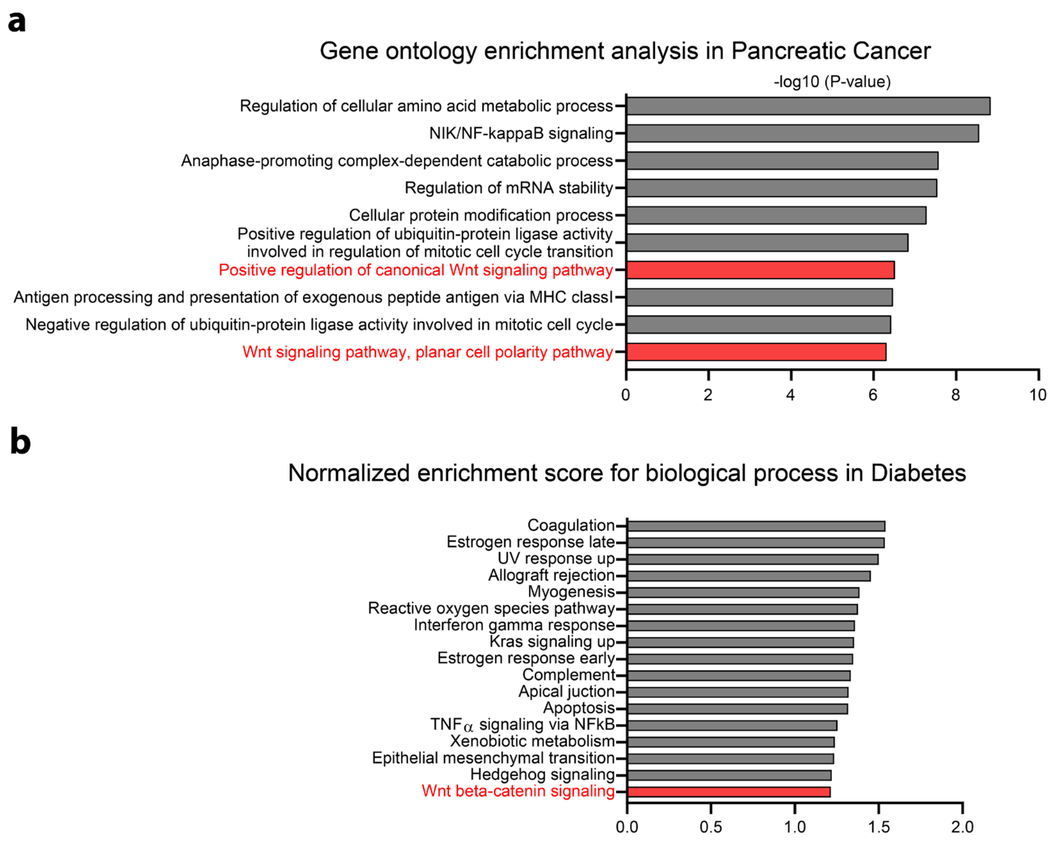

Supplementary Fig. 3 Geneset enrichment analyses of pancreatic cancer and diabetes

a Gene ontology enrichment analysis in pancreatic cancer. **b** Normalized enrichment score for biological process in diabetes.

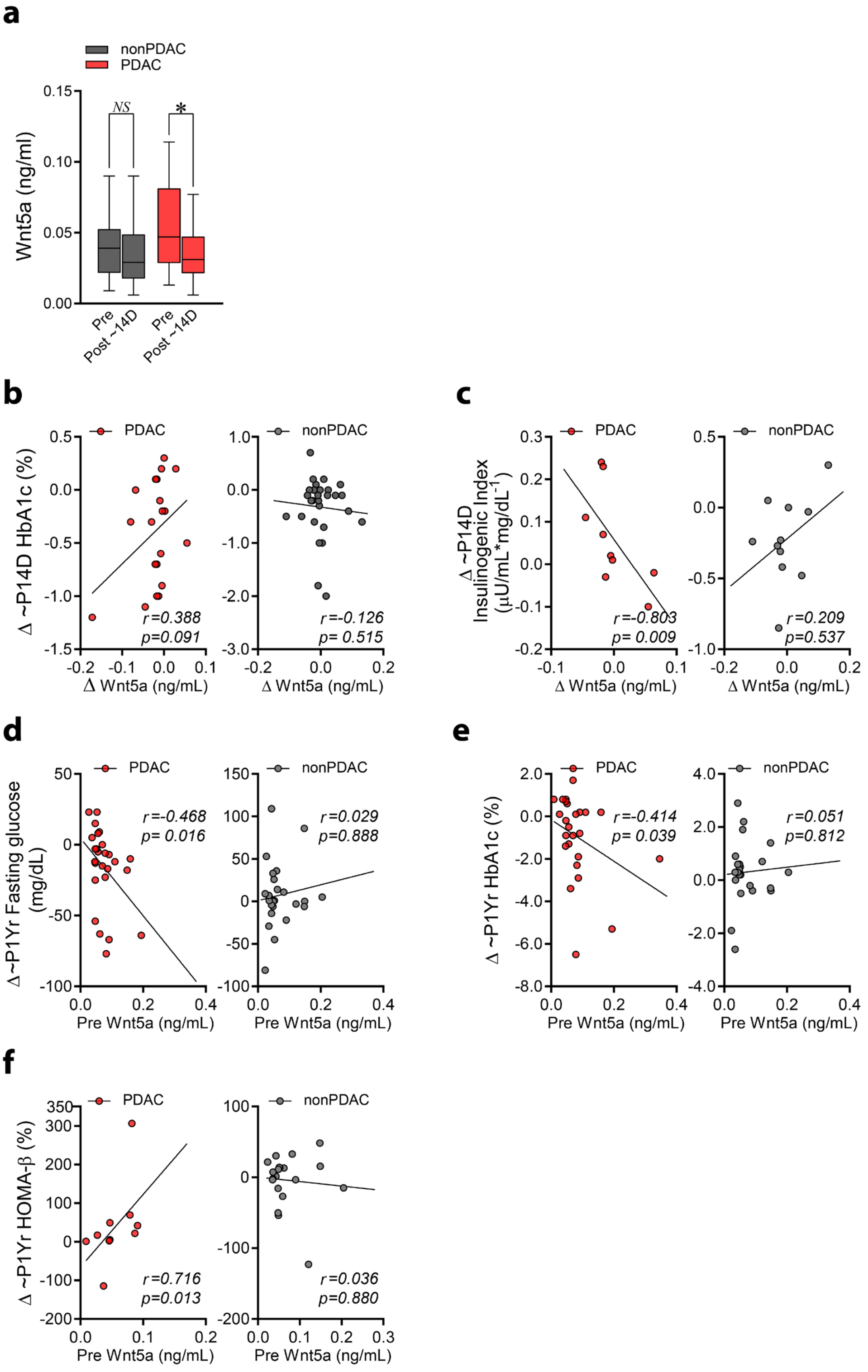

Supplementary Fig. 4 Plasma Wnt5a levels and its association with glucometabolic parameters after surgery

a Changes in plasma Wnt5a level between patients with and without PDAC before and 14 days after surgery. **b**–**c** Changes in plasma Wnt5a before and 14 days after surgery was calculated as [value at 14 days after surgery − value before surgery]. Correlation plots showing the associations between changes in plasma Wnt5a and changes in **b** HbA1c and **c** insulinogenic index before and 14 days after surgery in patients with and without PDAC. **d**–**f** Correlation plots showing the associations between pre-operative plasma Wnt5a and changes in **d** fasting glucose, **e** HbA1c, and **f** HOMA-β before and 1 year after surgery in patients with and without PDAC. Difference in each parameter was calculated as [Postoperative value − Preoperative value].

P* <0.05 by Wilcoxon signed-rank test (a**) or Spearman's rank correlation coefficient (**b**, **c**, **d**, **e**, and **f**); *NS*: statistically non-significant.

HbA1c, glycated hemoglobin A1c; HOMA-β, homeostasis model assessment for β-cell function; OP., operation; PDAC, pancreatic ductal adenocarcinoma.

-

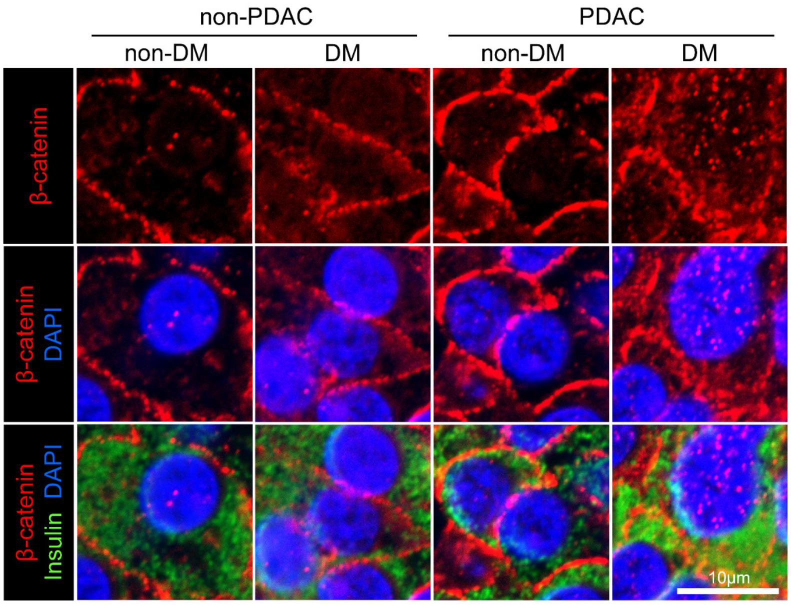

Supplementary Fig. 5 High-magnification immunofluorescence staining images for visualization of intracellular β-catenin localization in pancreas

Pancreatic tissue from the same site of resection margin was obtained from both PDAC and non-PDAC patients and then immunostained for β-catenin and insulin. Representative immunofluorescence staining images for β-catenin (red) and insulin (green) in PDAC and non-PDAC patients with and without DM. Magnification, 2800×. Scale bar, 10 µm.

DAPI, 4',6-diamidino-2-phenylindole; DM, diabetes mellitus; PDAC, pancreatic ductal adenocarcinoma

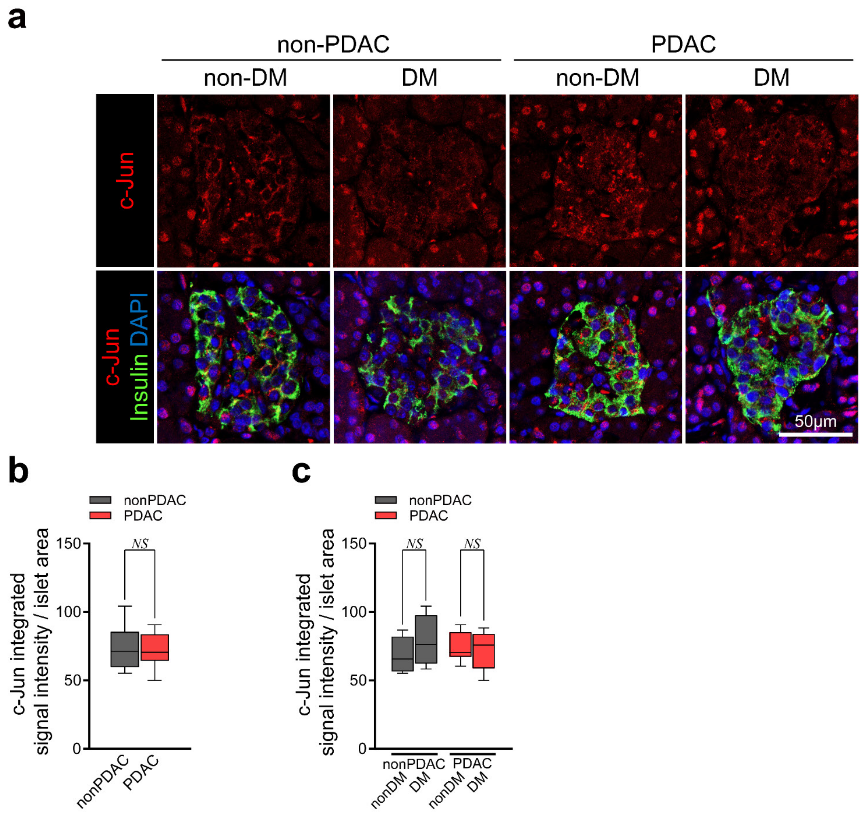

Supplementary Fig. 6. Islet c-Jun expression in relation to PDAC and diabetes status

a Representative immunofluorescence staining images for c-Jun (red) and insulin (green) in PDAC and non-PDAC patients with and without diabetes. Magnification, 400×. Scale bar, 50 μm. **b**–**c** The integrated signal intensity of c-Jun expression per islet area was calculated. **b** Differences of islet c-Jun expression between PDAC and non-PDAC groups. **c** Islet c-Jun expression according to diabetes status in PDAC and non-PDAC groups.

P* <0.05 by Student's *t*-test (b** and **c**); *NS*: statistically non-significant.

DAPI, 4',6-diamidino-2-phenylindole; DM, diabetes mellitus; PDAC, pancreatic ductal adenocarcinoma.

SUPPLEMENTARY TABLES

Supplementary Table 1. Comparison of the baseline characteristics in PDAC and non-PDAC groups

	Total (n=160)	non-PDAC group (n=88)	PDAC group (n=72)	*P*-values
Age (yr)	64 [57, 72]	63 [57,71]	65 [57,72]	0.644
Male (n, (%))	94 (58.8)	53 (60.2)	41 (56.9)	0.675
Pre-DM and DM (n, (%))	124 (77.5)	62 (70.5)	62 (86.1)	**0.018**
DM (n, (%))	75 (46.9)	35 (39.8)	40 (55.6)	**0.047**
New-onset diabetes (n, (%))§	40 (25.6)	17 (19.3)	23 (31.9)	**0.012**
New-onset or recently aggravated diabetes (n, (%))§	56 (35.0)	23 (26.1)	33 (45.8)	**0.009**
Acute or chronic pancreatitis (n, (%))	7 (4.6)	5 (6.0)	2 (2.9)	0.454
Hypertension (n, (%))	90 (56.3)	49 (55.7)	41 (56.9)	0.873
Cardiovascular disease (n, (%))	8 (5.0)	4 (4.5)	4 (5.6)	>0.999
Dyslipidemia (n, (%))	112 (70.0)	63 (71.6)	49 (68.1)	0.627
Systolic blood pressure (mmHg)	126 [111, 136]	127 [115, 136]	123 [110, 133]	0.358
Diastolic blood pressure (mmHg)	76 [70, 81]	76 [70, 82]	76 [70, 80]	0.536
Waist circumference (cm)	86.9 ± 9.5	86.8 ± 9.9	87.0 ± 9.0	0.921
Weight (kg)	63.4 ± 10.7	63.9 ± 11.2	62.8 ± 10.0	0.510
BMI (kg/m^2)	23.8 ± 3.0	23.3 ± 3.2	23.6 ± 2.8	0.600
2hr OGTT				

	Total	non-PDAC group	PDAC group	*P*-values
Glucose 0 min (mg/dL)	112 [101, 136]	106 [96, 119]	123 [106, 148]	**< 0.001**
Glucose 120 min (mg/dL)	220 [158, 318]	181 [143, 247]	300 [218, 349]	**< 0.001**
Glucose 0~120 min AUC	24285 [18885, 30195]	21758 [17689, 26779]	27600 [22838, 33173]	**0.001**
Insulin 0 min (μIU/mL)	6.4 [4.1, 11.0]	7.6 [4.3, 12.4]	6.0 [3.9, 10.3]	0.063
Insulin 120 min (μIU/mL)	34.7 [17.5, 63.9]	45.4 [21.8, 78.1]	27.7 [14.4, 48.9]	**0.026**
Insulin 0~120 min AUC	3589 [1843, 6095]	4679 [3192, 8284]	2366 [1192, 3652]	**< 0.001**
HbA1c (%)	5.8 [5.4, 6.9]	5.6 [5.2, 6.4]	6.2 [5.6, 8.1]	**< 0.001**
Fasting C-peptide (ng/mL)	2.25 [1.61, 3.71]	2.58 [1.78, 4.40]	2.00 [1.44, 2.84]	**0.013**
Fasting glucagon (pg/mL)	178 [106, 256]	185 [90, 287]	177 [108, 228]	0.468
Triglyceride (mg/dL)	133 [84, 191]	140 [83, 217]	127 [84, 161]	0.170
HDL-Cholesterol (mg/dL)	39 [31, 47]	37 [31, 45]	41 [32, 49]	0.115
LDL-Cholesterol (mg/dL)	112 [89, 140]	113 [90, 141]	108 [86, 141]	0.578
Total bilirubin (mg/dL)	0.9 [0.6, 2.4]	0.9 [0.6, 2.2]	0.1 [0.6, 2.6]	0.464
γ-glutamyl transferase (U/L)	565 [19, 257]	65 [19, 205]	67 [18, 335]	0.508
eGFR (CKD-EPI) (mL/min/1.73 m^2)	101.0 [88.0, 118.8]	98.5 [85.3, 117]	101.5 [93.3, 126.8]	0.070

**Homeostasis model assessment**	**Total (n=131)**	**non-PDAC group (n=72)**	**PDAC group (n=59)**	***P*-values**
HOMA-IR (mg/dL*μIU/mL)	1.9 [1.1, 3.2]	1.8 [1.1, 3.5]	1.9 [1.1, 3.1]	0.660
HOMA-β (%)	48.5 [27.4, 81.0]	63.0 [36.3, 95.1]	33.6 [23.1, 63.9]	**< 0.001**

**Insulin sensitivity and release indices**	**Total (n=83)**	**non-PDAC group (n=46)**	**PDAC group (n=37)**	***P*-values**

Matsuda index	4.9 [3.2, 7.6]	4.2 [3.2, 6.8]	6.4 [3.1, 8.1]	0.127
Insulinogenic index (μU/mL*mg/dL^{-1})	0.30 [0.07, 0.54]	0.39 [0.17, 0.63]	0.12 [0.04, 0.34]	**0.001**
Disposition index	0.93 [0.32, 2.30]	1.81 [0.56, 2.99]	0.49 [0.20, 1.47]	**0.001**
**Histological assessment for pancreas**	**Total (n=74)**	**non-PDAC group (n=47)**	**PDAC group (n=27)**	***P*-values**
Masson's trichrome-positive area of pancreas (%)	15 [3, 35]	12 [3, 24]	33 [8, 58]	**0.001**

Data are described as mean±standard deviation, median [interquartile range], or numbers (percentage). The *p*-values for the comparisons of continuous variables between PDAC versus non-PDAC groups were determined by either Student's *t*-test or Mann-Whitney *U*-test as appropriate. Fisher's exact test was applied for categorical variables, when more than 20% of categories had an expected frequency of <5; otherwise, the chi-square test was used. Statistically significant values are indicated in **Bold** (*P* <0.05).

BMI, body mass index; OGTT, oral glucose tolerance test; AUC, area under the curve; CKD-EPI, Chronic Kidney Disease Epidemiology Collaboration; DM, diabetes mellitus; eGFR, estimated glomerular filtration rate; HbA1c, glycated hemoglobin A1c; HDL-C, high-density lipoprotein cholesterol; LDL-C, low-density lipoprotein cholesterol; PDAC, pancreatic ductal adenocarcinoma. HOMA-IR, homeostatic model assessment of insulin resistance; HOMA-β, homeostasis model assessment of β-cell function.

[§]New-onset diabetes is defined as diabetes within less than 2 years before entry into the cohort and recently aggravated diabetes is defined as uncontrolled hyperglycemia within 1 year before entry into the cohort.

Supplementary Table 2. Changes in glucometabolic parameters at 14 days after PPPD

	non-PDAC group (n=58)			PDAC group (n=39)			*P*-values
	**Pre**	**Post 14d**	**Diff.**	**Pre**	**Post 14d**	**Diff.**	
BMI (kg/m^2)	24.2 ± 3.2	23.5 ± 3.2*	-1.0 [-1.7, -0.4]	23.4 ± 2.7	22.6 ± 2.7*	-0.8 [-1.3, -0.32]	0.129
2hr OGTT							
Glucose 0 min (mg/dL)	106 [96, 115]	113 [104, 136]*	8 [-4, 22]	120 [106, 152]#	127 [110, 149]	0 [-1.6, 16]	0.085
Glucose 120 min (mg/dL)	191 [151, 243]	174 [139, 223]	-23 [-54, 10]	297 [213, 352]#	199 [158, 274]*	-61 [-108, -30]	**0.002**
Glucose 0~120 min AUC	23985 ± 7667	19260 [17359, 24510]*	-3233 [-7714, 863]	28155 ± 8086	23235 [18180, 27225]*	-4335 [-7995, -2168]	0.311
Insulin 0 min (μIU/mL)	8.4 [4.8, 13.1]	4.8 [3.0, 7.6]*	-2.5 [-7.7, 0.0]	5.4 [3.8, 10.5]	4.7 [2.9, 6.6]*	-1.8 [-4.4, 0.0]	0.239
Insulin 120 min (μIU/mL)	43.5 [22.5, 74.6]	18.7 [11.5, 29.8]*	-23.1 [-42.4, -9.3]	25.3 [12.6, 41.7]#	14.4 [8.0, 24.8]*	-11.4 [-18.3, -2.1]	0.119
Insulin 0~120 min AUC	4346 [3190, 6224]	2084 [1073, 3541]*	-2103 [-3014, -810]	1344 [1017, 3564]#	1116 [801, 2787]	-239 [-1191, -84]	**0.002**
HbA1c (%)	5.5 [5.1, 6.2]	5.3 [5.0, 5.8]*	-0.1 [-0.5, 0.1]	6.5 [5.7, 7.5]#	5.8 [5.4, 6.7]*	-0.4 [-1.0, -0.1]	**0.015**
HOMA-IR (mg/dL*μIU/mL)	2.08 [1.31, 3.61]	1.38 [0.87, 2.15]*	-0.47 [-2.27, 0.06]	1.87 [1.19, 2.75]	1.39 [1.02, 2.20]*	-0.53 [-1.48, 0.06]	0.489
HOMA-β (%)	66.6 [38.8, 102.7]	40.7 [21.5, 52.0]*	-27.2 [-52.0, -2.6]	34.0 [21.5, 71.2]#	26.5 [16.6, 44.5]*	-12.4 [-28.9, -2.5]	0.091
Matsuda index	3.96 [3.21, 6.63]	8.58 [5.64, 15.12]*	3.60 [1.06, 8.74]	6.99 ± 3.71	8.86 [5.58, 15.89]*	2.13 [-1.37, 9.32]	0.588
Insulinogenic index (μU/mL*mg/dL^{-1})	0.38 [0.16, 0.62]	0.21 [0.08, 0.44]*	-0.10 [-0.28, 0.03]	0.10 [0.02, 0.36]#	0.09 [0.05, 0.39]	0.01 [-0.02, 0.07]	**0.018**

Disposition index	1.81 [0.61, 2.39]	1.31 [0.83, 3.09]	0.09 [-0.87, 0.99]	0.50 [0.17, 1.64][#]	1.09 [0.42, 1.95]*	0.58 [-0.09, 1.13]	0.190
Pancreas volume (cm^3)[†]	70.1 ± 25.8	37.6 [25.4, 49.8]*	-30.4 [-44.0, -19.7]	54.3 ± 20.7[#]	21.1 [13.2, 31.8]*,[#]	-25.9 [-39.4, -17.1]	0.318

Data are described as mean±standard deviation or median [interquartile range]. The *p*-values are for the comparisons of differences in the pre- versus 14 days post-surgery between PDAC versus non-PDAC groups, using either Student's *t*-test or Mann-Whitney *U*-test as appropriate. Statistically significant values are indicated in **Bold** (P <0.05).

*P <0.05 versus the pre-operative status in each group. [#]P <0.05 versus non-PDAC group.

[†]Pancreas volume were measured in 53 non-PDAC and 36 PDAC participants.

AUC, area under the curve; BMI, body mass index; HbA1c, glycated hemoglobin A1c; HOMA-IR, homeostatic model assessment of insulin resistance; HOMA-β, homeostasis model assessment of β-cell function; PPPD, pylorus-preserving pancreaticoduodenectomy; Pre, pre-operative status, Post 14d, post-operative status 14 days after surgery.

Supplementary Table 3. Changes in glucometabolic parameters 1 year after PPPD

	non-PDAC group (n=30)			PDAC group (n=28)			*P*-values
	Pre	Post 1yr	Diff.	Pre	Post 1yr	Diff.	
BMI (kg/m^2)	23.9 ± 3.5	23.3 ± 3.3*	-0.6 ± 1.3	23.5 ± 2.8	22.2 ±2.8*	-1.3 ± 1.9	0.108
2hr OGTT							
Glucose 0 min (mg/dL)	107 [96, 117]	105 [97, 118]	1 [-13, 9]	130 [107, 155]#	109 [94, 120]*	-17 [-59, -2]	**0.001**
Glucose 120 min (mg/dL)	188 [151, 246]	140 [110, 198]*	-40 [-96, -11]	309 [195, 362]#	187 [140, 236]*	-77 [-139, -36]	0.123
Glucose 0~120 min AUC	25133 ± 7967	17018 [16313, 23464]*	4808 [-6653, -1414]	31114 ± 6604	23685 [20479, 28095]	-1455 [-12465, 390]	0.954
Insulin 0 min (µIU/mL)	7.3 [4.9, 11.0]	6.9 [4.2, 8.1]	-0.4 [-4.4, 1.8]	4.7 [3.4, 7.6]	6.4 [3.9, 9.1]	1.0 [-1.4, 3.3]	0.225
Insulin 120 min (µIU/mL)	61.1 [25.9, 89.0]	24.5 [17.0, 39.7]*	-23.0 [-71.2, 0.9]	20.5 [12.6, 173.3]	26.9 [14.5, 36.7]	3.7 [-20.4, 8.8]	0.203
Insulin 0~120 min AUC	4995 [3240, 8838]	3794 [3382, 5180]	-603 [-2999, 178]	1212 [954, 5449]#	2795 [1508, 6996]	998 [-915, 2412]	0.096
HbA1c (%)	5.5 [5.0, 6.4]	5.8 [5.4, 6.2]	0.2 [-0.2, 0.6]	6.5 [5.7, 8.1]#	6.0 [5.4, 6.7]*	-0.3 [-1.0, 0.2]	**0.007**
HOMA-IR (mg/dL*µcIU/mL)	3.20 [1.82, 5.78]	2.39 [1.72, 3.68]	0.55 [-0.03, 2.00]	2.70 [1.96, 4.80]	2.56 [1.62, 2.89]	0.72 [-0.04, 1.99]	0.731
HOMA-β (%)	60.9 [39.3, 89.7]	58.7 [38.5, 77.1]	-2.0 [-17.4, 13.8]	30.1 [13.8, 48.5]#	51.4 [18.3, 87.6]*	17.1 [4.0, 41.8]	**0.017**
Matsuda index	3.40 [2.18, 5.90]	5.95 ± 2.66	1.41 ± 2.51	6.59 [1.45, 7.39]	5.38 ± 3.47	0.20 ± 3.02	0.351
Insulinogenic index ($\mu U/mL*mg/dL^{-1}$)	0.38 [0.17, 0.78]	0.51 [0.23, 0.91]	0.07 [-0.16, 0.16]	0.06 [0.03, 0.37]#	0.15 [0.06, 0.55]	0.03 [-0.01, 0.32]	0.688

Disposition index	1.87 [0.66, 4.69]	2.50 [1.18, 6.12]*	0.87 [0.32, 2.78]	0.47 [0.28, 1.05]	0.74 [0.63, 1.83]	0.34 [0.19, 0.69]	0.083

Data are described as mean±standard deviation or median [interquartile range]. The *p*-values are for the comparisons of differences in the pre- versus 1 year post-surgery between PDAC versus non-PDAC groups, using either Student's *t*-test or Mann-Whitney *U*-test as appropriate. Statistically significant values are indicated in **Bold** (P <0.05).

*P <0.05 versus the pre-operative status in each group. #P <0.05 versus non-PDAC group.

BMI, body mass index; OGTT, oral glucose tolerance test; AUC, area under the curve; HbA1c, glycated hemoglobin A1c; HOMA-IR, homeostatic model assessment of insulin resistance; HOMA-β, homeostasis model assessment of β-cell function; PPPD, pylorus-preserving pancreaticoduodenectomy; Pre, pre-operative status, Post 1yr, post-operative status in 1 year after surgery.

Supplementary Table 4. Correlation between preoperative plasma Wnt5a levels and baseline glucometabolic parameters

	non-PDAC group (n=53)		PDAC group (n=47)	
	r	*p*-values	*r*	*p*-values
Age (years)	-0.116	0.408	0.059	0.692
BMI (kg/m^2)	-0.002	0.993	-0.173	0.444
Duration of diabetes (years)	-0.110	0.431	0.185	0.214
PDAC tumor size (cm)	–	–	**0.351**	**0.019**
2hr oral glucose tolerance test				
Glucose 0 min (mg/dL)	-0.224	0.107	-0.046	0.759
Glucose 120 min (mg/dL)	-0.050	0.807	0.090	0.648
Glucose 0~120 min AUC	-0.076	0.736	**0.489**	**0.039**
Insulin 0 min (μIU/mL)	-0.328	0.028	-0.237	0.126
Insulin 120 min (μIU/mL)	-0.053	0.814	-0.024	0.916
Insulin 0~120 min AUC	0.022	0.922	**-0.499**	**0.030**
HbA1c (%)	-0.070	0.623	**0.379**	**0.009**
Matsuda index	0.158	0.483	-0.107	0.644
HOMA-IR (mg/dL*μIU/mL)	-0.350	0.019	-0.191	0.220
HOMA-β (%)	-0.215	0.156	-0.316	**0.044**
Insulinogenic index (μU/mL*mg/dL^{-1})	0.076	0.736	**-0.649**	**0.005**
Disposition index	0.030	0.895	-0.312	0.158
CEA (ng/mL)	-0.172	0.218	0.259	0.079
CA 19-9 (U/mL)	-0.072	0.625	**0.419**	**0.005**

Data are presented as Spearman's rank correlation coefficient (*r*).

Supplementary Table 5. Correlation between pancreatic β-catenin expression levels and baseline glucometabolic parameters

	non-PDAC group (n=19)		PDAC group (n=19)	
	r	*p*-values	*r*	*p*-values
Age (years)	-0.271	0.261	0.311	0.195
BMI (kg/m^2)	-0.154	0.652	-0.089	0.849
Duration of diabetes (years)	0.054	0.825	0.346	0.147
PDAC tumor size (cm)	–	–	**0.532**	**0.034**
2hr oral glucose tolerance test				
Glucose - 0 min (mg/dL)	0.119	0.628	**0.510**	**0.037**
Glucose - 120 min (mg/dL)	0.327	0.185	**0.698**	**0.008**
Glucose - 0~120 min AUC	0.174	0.489	**0.664**	**0.026**
Insulin - 0 min (μIU/mL)	-0.107	0.663	-0.348	0.187
Insulin - 120 min (μIU/mL)	0.267	0.284	**-0.664**	**0.026**
Insulin - 0~120 min AUC	0.131	0.604	**-0.727**	**0.011**
HbA1c (%)	0.278	0.249	**0.736**	**< 0.001**
Matsuda index	-0.112	0.657	0.545	0.083
HOMA-IR (mg/dL*μIU/mL)	-0.109	0.658	-0.186	0.431
HOMA-β (%)	-0.074	0.764	**-0.557**	**0.031**
Insulinogenic index (μU/mL*mg/dL^{-1})	-0.032	0.900	**-0.736**	**0.010**
Disposition index	-0.011	0.964	**-0.806**	**0.005**
CEA (ng/mL)	0.105	0.668	0.312	0.193
CA 19-9 (U/mL)	0.277	0.282	-0.047	0.852
Plasma Wnt5a (ng/mL)	0.282	0.272	**0.547**	**0.035**

Data are presented as Spearman's rank correlation coefficient (*r*).
